# Predictiveness of the Human-CYP3A4-Transgenic Mouse Model (Cyp3aXAV) for Human Drug Exposure of CYP3A4-Metabolized Drugs

**DOI:** 10.3390/ph15070860

**Published:** 2022-07-13

**Authors:** David Damoiseaux, Wenlong Li, Alejandra Martínez-Chávez, Jos H. Beijnen, Alfred H. Schinkel, Alwin D. R. Huitema, Thomas P. C. Dorlo

**Affiliations:** 1 Department of Pharmacy & Pharmacology, The Netherlands Cancer Institute, 1066 CX Amsterdam, The Netherlands; d.damoiseaux@nki.nl (D.D.); j.beijnen@nki.nl (J.H.B.); a.huitema@nki.nl (A.D.R.H.); 2 Division of Pharmacology, The Netherlands Cancer Institute, 1066 CX Amsterdam, The Netherlands; w.li@nki.nl (W.L.); d.martinez@nki.nl (A.M.-C.); a.schinkel@nki.nl (A.H.S.); 3 Utrecht Institute of Pharmaceutical Sciences, Utrecht University, 3584 CG Utrecht, The Netherlands; 4 Department of Clinical Pharmacy, University Medical Center Utrecht, Utrecht University, 3584 CX Utrecht, The Netherlands; 5 Department of Pharmacology, Princess Máxima Center for Pediatric Oncology, Utrecht University, 3584 CS Utrecht, The Netherlands

**Keywords:** population pharmacokinetics, extrapolation, first-in-human dose, Human-CYP3A4-transgenic mouse, CYP3A4-metabolized small-molecule drugs

## Abstract

The extrapolation of drug exposure between species remains a challenging step in drug development, contributing to the low success rate of drug approval. As a consequence, extrapolation of toxicology from animal models to humans to evaluate safe, first-in-human (FIH) doses requires high safety margins. We hypothesized that a human-CYP3A4-expressing transgenic (Cyp3aXAV) mouse is a more predictive model for human drug exposure of CYP3A4-metabolized small-molecule drugs. Population pharmacokinetic models based on wild-type (WT) and Cyp3aXAV mouse pharmacokinetic data of oral lorlatinib, brigatinib, ribociclib and fisogatinib were allometrically scaled and compared to human exposure. Extrapolation of the Cyp3aXAV mouse model closely predicted the observed human exposure for lorlatinib and brigatinib with a 1.1-fold and 1.0-fold difference, respectively, compared to a 2.1-fold and 1.9-fold deviation for WT-based extrapolations of lorlatinib and brigatinib, respectively. For ribociclib, the extrapolated WT mouse model gave better predictions with a 1.0-fold deviation compared to a 0.3-fold deviation for the extrapolated Cyp3aXAV mouse model. Due to the lack of a human population pharmacokinetic model for fisogatinib, only median maximum concentration ratios were calculated, resulting in ratios of 1.0 and 0.6 for WT and Cyp3aXAV mice extrapolations, respectively. The more accurate predictions of human exposure in preclinical research based on the Cyp3aXAV mouse model can ultimately result in FIH doses associated with improved safety and efficacy and in higher success rates in drug development.

## 1. Introduction

Of all investigated drugs, anticancer drugs have the lowest approval success rate [1]. One of the reasons is the lack of preclinical models, both in vitro assays and in vivo animal models, which are able to accurately predict human pharmacokinetics (PK) and the pharmacodynamics of new compounds [2,3]. Typically, animal and in vitro metabolic and transporter models are used preclinically to approximate human drug exposure as closely as possible and to inform the first-in-human (FIH) dose [4]. Although in vitro studies are useful for characterizing what enzymes and transporters are involved in the PK of a drug, in vivo models also take into account factors such as blood flow, tissue distribution and others involved in the absorption, distribution, metabolism and excretion (ADME) of drugs, making them more suitable for quantitative predictions. Still, finding an appropriate animal model to give accurate predictions remains challenging in the context of predicting FIH doses [5,6,7]. This is why organizations such as the World Health Organization (WHO) and European Medicines Agency (EMA) recommend setting a starting dose with a 10-fold safety margin over the severely toxic dose in 10% of animals (STD 10), which can be subdivided into a 4-fold margin for toxicokinetics and 2.5-fold margin for toxicodynamics [8,9].

Selection of the most representative species is crucial and should be carefully considered [7]. Humans and other species differ in many anatomical, physiological and biochemical aspects. Hence, the species that shares with humans the most characteristics that are of importance in the ADME of the relevant compound should be chosen. One of the hurdles is the inconsistency of metabolizing enzymes between species. In, for instance, mice, the Cyp3a enzymes have no clear orthologous pairs with that of humans; the four human Cytochromes P450 (CYP)3As (CYP3A4, −5, −7, and −43) and the eight full-length mouse Cyp3as do not match [10]. However, these proteins show extensive overlap in tissue distribution and substrate specificity. The combined functions of all the wild-type (WT) mouse Cyp3as are very likely to correspond to the combined function of all the human CYP3As. Nevertheless, the WT mouse is probably not the most suitable model to investigate CYP3A4 metabolism. To overcome this issue, a human-CYP3A4-transgenic (Cyp3aXAV) mouse model was developed to assess the effect of CYP3A4 in a qualitative manner [11]. The Cyp3aXAV is a mouse model that is knocked out for mouse Cyp3a and expresses human CYP3A4 in both the liver and intestines. This might be a more representative animal model for the PK of CYP3A4-metabolized compounds in humans compared to the WT mouse model [12,13]. We hypothesized that this murine model can also enable improved quantitative predictions of human exposure.

In interspecies extrapolation, simple allometric scaling based on weight or body surface area alone is often not expected to give adequate extrapolations [14]. Amongst other elements, the mechanistic understanding of underlying PK processes is unaccounted for, as well as physiological differences in enzymes and transporters between species. Modeling approaches can be used to optimize the extrapolation by accounting for these differences as much as possible. The most common model approaches used to perform interspecies extrapolation with allometric scaling are physiologically-based pharmacokinetic and population PK modeling [15,16,17]. Compartmental population PK modeling can be useful for revealing underlying PK processes that are known to be different between species and can cause potential difficulties in extrapolation. More importantly, it allows a more advanced use of allometric scaling by the scaling of primary PK parameters instead of secondary PK parameters [18]. Sparse and heterogeneous sampling can be used for this approach, although sufficient and informative samples in the index species must be available.

We aimed to investigate the predictiveness of a quantitative extrapolation of the murine Cyp3aXAV model to humans compared to that of the WT mouse model for CYP3A4-metabolized compounds. We used an acceptance criterion of a 2-fold difference in terms of exposure and assessed the overall predictiveness of the shape of the extrapolated human PK curve. We investigated this for four different CYP3A4 substrates: lorlatinib, brigatinib, fisogatinib and ribociclib, for which PK data from the murine Cyp3aXAV and WT models were available.

## 2. Results

### 2.1. Mouse Population PK Models

**Figure 1:**
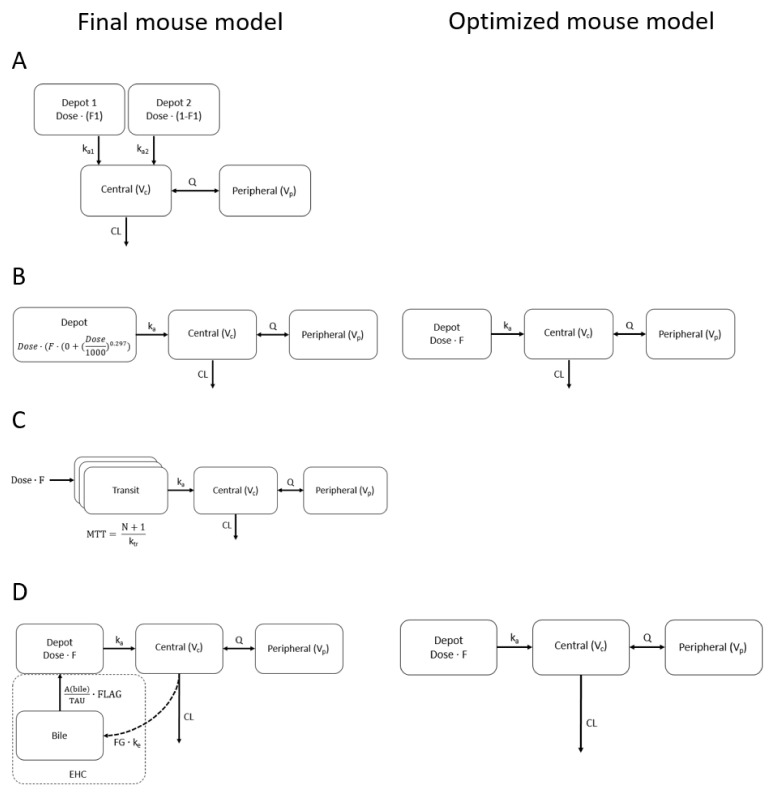
Schematic representation of (**A**) lorlatinib [18], (**B**) brigatinib, (**C**) fisogatinib and (**D**) ribociclib models in mice. The final mouse models represent the mouse models that best fitted the data, and the optimized mouse model (if applicable) was added if the best-fitted model contained properties that were redundant for extrapolation; in the optimized mouse model, these properties were left out. Mouse models are elaborated on in the Supplementary Materials.

**Figure 2:**
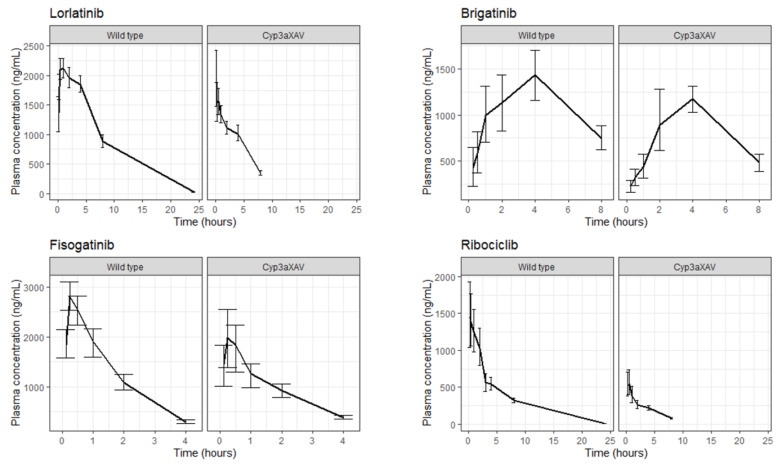
Median concentration time plots with standard deviation of lorlatinib, brigatinib, fisogatinib and ribociclib for wild-type and Cyp3aXAV mice.

A total of 658 (of which 112 were intravenous), 366, 414 and 270 plasma concentrations from 94, 61, 71 and 49 mice in six, two, three and two experiments were modeled using a compartmental population PK modeling approach for lorlatinib, brigatinib, ribociclib and fisogatinib, respectively. The development and evaluation of these models are described in more depth in Supplementary Materials. The models that best fitted the data were a two-compartment model with dual first-order absorption for lorlatinib, a two-compartment model with an exponential dose effect on F for brigatinib, a one-compartment model with transit compartments and an enterohepatic circulation model (EHC) for fisogatinib and a two-compartment model with an EHC for ribociclib (Figure 1 final mouse model). In Figure 2, concentration–time curves for WT and Cyp3aXAV mice are shown for all four drugs. Based on these plots, Cyp3aXAV mice, which express human CYP3A4, seem to have a more active CYP3A4 metabolism than the WT mice, which is explained in the model by the covariate effects observed on the parameters CL and F. Significant covariates (dOFV < −6.64 (*p* < 0.01, one degree of freedom)) identified for Cyp3aXAV mice relative to WT mice were 1.34-fold higher CL (relative standard error (RSE), 5%) and 0.71-fold lower F (RSE, 7%) for lorlatinib; 1.63-fold higher CL (RSE, 13%) for brigatinib; 3.53-fold higher CL (RSE, 8%) for ribociclib; and 0.61-fold lower CL (RSE, 6%) and 0.57-fold lower F (RSE, 10%) for fisogatinib (Supplementary Materials, Table S2). Removal of redundant model properties (reason for removal explained in Section 2.2.) resulted in the following final models for mouse-to-human translation: a two-compartment model with a dual first-order absorption for lorlatinib, a two-compartment model for brigatinib, a two-compartment model with transit compartments for fisogatinib and a two-compartment model for ribociclib (Figure 1). Significant covariates (dOFV < −6.64 (*p* < 0.01, one degree of freedom)) identified in these final models for mouse-to-human translation for the Cyp3aXAV strain relative to WT strain were 1.34-fold higher CL (RSE, 5%) and 0.71-fold lower F (RSE, 7%) for lorlatinib; 1.91-fold higher CL (RSE, 9%) for brigatinib; 0.30-fold lower F (RSE, 8%) for ribociclib; and 0.61-fold lower CL (RSE, 6%) and 0.57-fold lower F (RSE, 10%) for fisogatinib (Supplementary Materials, Table S2).

### 2.2. Redundant Model Properties

**Figure 3:**
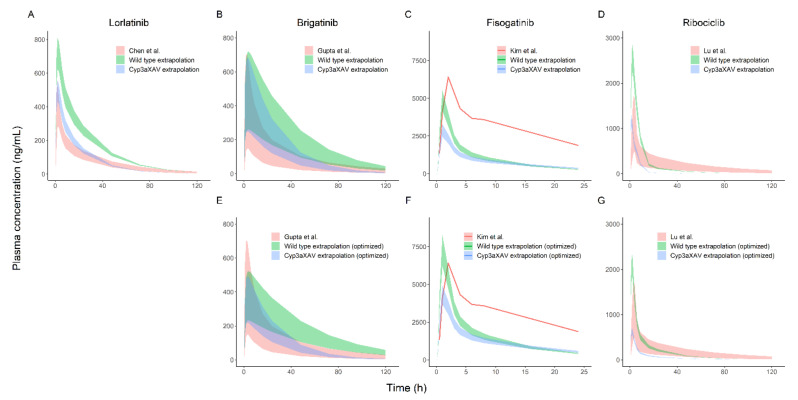
Plotted here are the 80% visual predictive intervals of the simulations for (**A**) lorlatinib, (**B**,**E**) brigatinib, (**C**,**F**) fisogatinib and (**D**,**G**) ribociclib of the model from literature (Chen et al. [20], Gupta et al. [21], Kim et al. [22] and Lu et al. [23]) and the extrapolated models for wild-type and Cyp3aXAV mice for final mouse models (**A**–**D**) and optimized mouse models (if applicable) (**E**–**G**). Standard human doses of 100, 180, 600 and 600 mg were simulated for lorlatinib, brigatinib, ribociclib and fisogatinib, respectively. Visual predictive intervals consisted of 500 simulations for the model from literature and one simulation for the extrapolated models. Simulations were from 0 to 120 h after dose, except for fisogatinib, where only human data up to 24 h after dose were available.

We aimed to use mouse PK models for extrapolation to human that were transparent and straightforward. EHC and exponential dose effect on F were considered to be redundant model properties because of limited physiological rationale in humans and were omitted from the models. Presence of an EHC for a compound in mice does not necessarily indicate that an EHC is identifiable in humans [19]. Secondly, implementing EHC in the ribociclib model in mice had minimal influence on the overall concentration–time curve, although it resulted in a significantly better fit (dOFV of −45 (three degrees of freedom, *p* < 0.005)). Nevertheless, the effect of these redundant model properties on human extrapolations was evaluated. Differences in extrapolations between models with and without (optimized mouse model) redundant model properties are visualized in Table 1 and Table 2 and Figure 1B,D and Figure 3B,D,E,G. The dose effect on F in the final mouse model of brigatinib (Figure 3B and Table 1) led to an over-prediction of the AUC_inf_ compared to clinical data which was not observed in the for translation-optimized mouse models (Figure 3E), indicating that dose effect on F indeed might not be present or is present to a lesser extent in humans. The best-fitted model for ribociclib included an EHC (Figure 3D), resulting in an over-predicted CL compared to the models without an EHC, which were more in line with the clinical data (Figure 3G).

### 2.3. AUC_inf_, C_max_ and Prediction Interval Comparison

The fold change between the median AUC_inf_ and C_max_ for WT- and Cyp3aXAV-extrapolated mice compared to clinical data is presented in Table 1 and Table 2, and the median AUC_inf_ and C_max_ itself and their 80% prediction intervals are shown in Figure 3A,E,F,G. Median AUC_inf_ ratios for Cyp3aXAV-extrapolated mice fell within the 0.5–2-fold criterion for lorlatinib (1.1-fold) and brigatinib (1.0-fold). This was substantiated by the extrapolation of the Cyp3aXAV mice for both lorlatinib and brigatinib, which had largely overlapping 80% prediction intervals (Figure 3A,E). For the WT mice, extrapolated median AUC_inf_ ratios were higher, with brigatinib just under the upper criterion level (1.9-fold) and lorlatinib just exceeding it (2.1-fold). Median C_max_ ratios in all extrapolations met the 0.5–2-fold criterion but only just for the WT-extrapolated mice for lorlatinib (2.0-fold). This contributed to the less adequate overlap of the 80% prediction interval of the extrapolation of WT mice for lorlatinib (Figure 3A). The less adequate overlap of the 80% prediction interval of the extrapolation of WT mice for brigatinib originated from a particular underestimation of the CL (Figure 3E). For ribociclib, both the 80% prediction intervals of WT and Cyp3aXAV mice extrapolation (Figure 3G) showed an over-prediction of the CL, resulting in an underestimated systemic exposure for Cyp3aXAV-extrapolated mice with a median AUC_inf_ ratio of 0.3-fold. For the WT-extrapolated mice, on the other hand, the median AUC_inf_ ratio (1.0-fold) met the criterion due to a higher F (median C_max_ ratio of 1.9-fold), compensating for the over-predicted CL. As predicted, the 80% prediction intervals of WT and Cyp3aXAV mice extrapolations for fisogatinib resulted in an under-prediction of F (Figure 3C), which was supported by the median C_max_ ratios of 0.8 and 0.5 for the WT and Cyp3aXAV mice extrapolations, respectively. This under-prediction was partially corrected for with the unionized fraction of fisogatinib in the mouse stomach, resulting in a lower F compared to in humans (optimized mouse model, Figure 3F) and resulting in increased median C_max_ ratios of 1.0 and 0.6 for WT and Cyp3aXAV mice extrapolations, respectively. Furthermore, the 80% prediction intervals of WT and Cyp3aXAV mice extrapolations for fisogatinib (Figure 3F) also showed an over-prediction of the CL. Due to the lack of a clinical population PK model for fisogatinib, no median AUC_inf_ ratios could be calculated.

## 3. Discussion

Extrapolation from the Cyp3aXAV mouse model closely predicted the in-human, observed secondary PK parameters for the compounds lorlatinib and brigatinib, which fell within the predefined 0.5–2-fold margin. The Cyp3aXAV mouse models also provided an adequate prediction of the PK profile based on the overlapping prediction intervals (Figure 3A,E). The extrapolated WT mouse model, on the other hand, did not optimally predict the human PK for lorlatinib and brigatinib, with brigatinib only just falling within the predefined 0.5–2-fold margin. Approximations of human PK for ribociclib and fisogatinib with the Cyp3aXAV model were not as accurate as for lorlatinib and brigatinib. Misspecifications that stood out were the under-prediction of F for fisogatinib and the slight over-prediction of CL in the extrapolations for both compounds (Figure 3F,G). However, predictions of other PK parameters were reasonable and only minimally outside the predefined 0.5–2-fold margin. Although the WT model gave slightly better predictions for ribociclib, similar over-predictions of the CL were observed. This indicates that the Cyp3aXAV model was an appropriate model to predict CYP3A4 metabolism, and misspecifications probably emerged from other inconsistent PK properties between human and mice.

The ADME of humans and mice differ with regard to the metabolic rates of the CYP3A enzyme family and other enzymes. Humans and mice differ in many anatomical, physiological and biochemical aspects [24]. It is hard to pinpoint all differences between human and mice PK that result in the misspecifications of the predictions of ribociclib and fisogatinib. In Table 3, the properties of the different drugs are summarized to identify explanatory factors for differences in prediction accuracy between compounds. The basic pK_a_ of fisogatinib is a potential explanation for the under-predicted F when extrapolating to humans. Incorporating the ionized fraction of fisogatinib in the stomach as an effect on F partially corrected for the under-prediction (Figure 3C,F). No drug properties could be identified that could explain why the CL of brigatinib and lorlatinib is more predictable than that of ribociclib and fisogatinib. Nevertheless, the prediction accuracy of secondary PK parameters with this method was remarkable for all four compounds since making accurate predictions of human exposure is still very challenging, and a 0.5–2-fold margin is a strictly set requirement in terms of interspecies extrapolation.

Cyp3aXAV- and WT-mouse-extrapolated population PK models were developed using the available data of the available knock-out and human expression of various transporters and metabolic enzymes. Physiologically plausible covariates were applied to relevant PK parameters to describe the effect of the different genetic modifications in the strains (Supplementary Materials). Covariate effects on the Cyp3aXAV mouse strain were found in either or both CL and F for all four compounds. This suggests that the human CYP3A4 in Cyp3aXAV mice is a more potent metabolizer of these compounds compared to the eight full-length mouse Cyp3as in WT mice, the combined function of which is expected to correspond to the combined function of all the human CYP3As (Figure 2). Logically, with a higher metabolizing rate, a higher CL and lower F are expected. In contrast, the covariate effects of Cyp3aXAV we found for fisogatinib were a 0.61 fold lower CL and a 0.57 fold lower F. This could be due to estimating two intertwined parameters such as CL and F. Overall, the estimated exposure was still found to be lower for Cyp3aXAV compared to WT. Furthermore, due to a lack of intravenous data for brigatinib, ribociclib and fisogatinib, no absolute F could be estimated for WT and only the relative F for Cyp3aXAV.

The Cyp3aXAV and WT mice population PK models were allometrically scaled to humans. Scaling was applied to all CL and volume of distribution parameters. Notable differences in study design between human and mice were evaluated and corrected for in the model if necessary. To prevent unnecessary complexity of the models, the aim was to incorporate as few and as transparent corrections as possible. The difference in route of administration, solution through gavage in the stomach of the mice versus tablet per os in humans, was corrected for by introducing a zero-order release or extended mean transit time to mimic the dissolution time of the tablet. Furthermore, no between-subject variability of parameters was extrapolated from mice to human. Our aim was to give an accurate prediction of the trends in exposure in humans and not the variation that can be expected. Nevertheless, we did incorporate the variation of the human models from literature to find out if at least a part of the population was described by the predictions of the extrapolated mouse models (Figure 3). Lastly, the dose effects on F and EHC in the mouse models of brigatinib and ribociclib were removed because these model features could not be extrapolated using the allometric scaling approach or were expected to be irrelevant for extrapolation (Figure 3B,D). The parameters involved in the EHC process do not necessarily follow the rules of allometry due to likely differences in physiology, and unscaled parameters are almost certainly different compared to the parameters you can expect in humans [19]. Nevertheless, interspecies extrapolation of models with an EHC is not impossible, as is shown by Kim et al., in whose study a model, including an EHC, was developed, and allometric scaling between species in combination with scaling of bile flow kinetic parameters was assessed [25]. However, we wanted to avoid introducing unnecessary complexity in the extrapolated models and deemed it better to simplify the models by removing these redundant model properties and re-estimating the model parameters.

Development of a population PK model with human-CYP3A4-expressing mice provided better knowledge about what PK parameters were altered by this enzyme, which allowed for a more evidence-based approach for animal-to-human extrapolation regarding compounds metabolized by this enzyme. The extrapolated mouse models provided a number of applications for future studies. The evidence-based extrapolation can be valuable for improving our understanding and interpretation of the role this enzyme might play in drug–drug interactions. Extrapolated mouse models can also be used to improve the designs of FIH trials. The International Counsel for Harmonization guideline for nonclinical evaluation for anticancer pharmaceuticals (S9) states that, currently, the common approach for the selection of the FIH dose is to set a start dose at 1/10 of the severely toxic dose in 10% of animals (STD 10) in rodents on a mg/m^2^ basis. If a non-rodent is the most appropriate species, then 1/6 of the highest non-severely toxic dose is considered an appropriate starting dose. This is a dose where no lethality, life-threatening toxicities or irreversible findings are presented [9]. In short, the FIH dose is empirically approximated based on only toxicity in preclinical models. We demonstrated that human PK of small-molecule anticancer drugs that are metabolized by CYP3A4 can be reasonably approximated using the Cyp3aXAV mouse model. This creates new opportunities that can contribute to more substantiated and evidence-based FIH dosage of CYP3A4-metabolized compounds, e.g., linking toxicity and pharmacodynamic effects to drug levels and providing a better understanding of which dose and dosing regimen is likely to be most appropriate. Furthermore, different sampling regimens can be simulated with the developed models to find optimal sampling designs for FIH trials to obtain the most informative data for determining human PK, as well as other study-design-related issues such as power analyses.

## 4. Methods

### 4.1. Data

A selection was made of four small-molecule anticancer drugs which are CYP3A4 substrates for which mice PK data are available in our research group from >99% FVB genetic background mice (both WT and Cyp3aXAV strains). All plasma concentration-time data used to develop the mouse models were generated in 5 previously published studies in mice receiving oral and intravenous lorlatinib and mice receiving oral brigatinib, ribociclib and fisogatinib [26,27,28,29,30]. An overview of the PK data and population PK model development for each of these compounds in the various mouse models is available in Supplementary Materials (Tables S1 and S2, Figures S1 and S2). Human plasma concentrations were simulated using human clinical population PK models from literature. The used models were a 2-compartment base model with auto-induction of CL and sequential zero-first order absorption for lorlatinib and a 3-compartment model with transit absorption for brigatinib based on data from both healthy volunteers and cancer patients. For ribociclib, a 2-compartment model with delayed zero-order oral absorption and first-order clearance (CL) from the central compartment based on cancer patients was used [20,21,23]. Lastly, for fisogatinib, no population PK model was available, and plasma concentrations were directly obtained from a FIH trial in patients with hepatocellular carcinoma using Plot Digitizer [22,31].

### 4.2. Population PK Models for Cyp3aXAV and WT Mouse Strains

Population PK models for lorlatinib, brigatinib, fisogatinib and ribociclib in mice were developed. One- and two-compartment and different absorption models were evaluated (Supplementary Materials). To account for differences between WT and Cyp3aXAV mice, strain was evaluated as a covariate on physiologically plausible parameters. Since the Cyp3aXAV mouse expresses human CYP3A4, of which lorlatinib, brigatinib, fisogatinib and ribociclib are known substrates, in the liver and intestines, differences in CL and bioavailability (F) were evaluated. Difference in objective function values (dOFVs) following a chi-squared distribution was considered significant for hierarchical models when <−6.64 (*p* < 0.01, 1 degree of freedom).

At last, PK processes implemented in the final mouse models were evaluated for their physiological relevance in humans and appropriateness for extrapolation using allometric scaling. PK processes that were thought to be redundant for the extrapolation were omitted from the final mouse models. Thereafter, parameters were re-estimated to obtain the best fit without the redundant model properties, resulting in the optimized mouse models for mouse-to-human translation (Supplementary Materials).

### 4.3. Extrapolation

All models were scaled to human by using standard allometric scaling of typical CL and volumes of distribution parameters as estimated based on the mouse data [32].

(1) $$CL_{human,i}=\theta_{mouse,pop\;CL}\cdot {\left(\frac{BWT}{30}\right)}^{0.75}$$

(2) $$V_{human,i}=\theta_{mouse,pop\;V}\cdot {\left(\frac{BWT}{30}\right)}^{1}$$

where *CL_human,i_* and *V_human,i_* are the bodyweight-corrected estimates in a human individual for *CL* and distribution volume, respectively. θ*_mouse,pop_* represents the population estimate for the relevant parameter in mice, and BWT represents the human individual bodyweight (gram), which is divided by the median weight (30 g) of the mouse population.

Between-subject variability was excluded from the extrapolation as variability in PK between mice was not considered representative of variability in PK in the human population. Mice with a >99% FVB genetic background are bred to have homogeneity in ADME-related processes to produce constant results and are, in this way, not comparable to the heterogeneity of the processes that we can expect in the human population. Within-subject (residual) variability was also excluded from the extrapolation as unexplained variability originating from, e.g., experimental errors and model misspecification, most likely differed between studies performed in mice and humans.

Notable differences in study design between mice and humans were corrected in the model. Mice received their dosage as a solution by gavage into the stomach, as opposed to tablets for humans [33]. As a correction, a zero-order release of the dose of the relevant tablets in the depot compartment using in vitro dissolution times (for >95%) of 45, 45 and 30 min for lorlatinib [34], brigatinib [35] and ribociclib [36], respectively, was added to correct for the route of administration. For fisogatinib, no dissolution profiles were publicly available, but, since it concerns an immediate-release formulation comparable to the other 3 compounds, we assumed a similar dissolution time of 45 min. Furthermore, the zero-order release to mimic the dissolution time was not an option for fisogatinib because of the way the transit absorption was modeled; as an alternative, we chose to add 0.375 h to the mean transit time parameter. In Table 3, the properties of the different drugs are summarized to identify explanatory factors for the differences between mice and humans. Fisogatinib’s basic pK_a_ of 3.79 might have resulted in an under-prediction of F in mice compared to humans. The normal murine gastric pH is 3–4, declining to an intestinal pH ~ 5 [37], whereas the human gastric pH is 1–2.5, and intestinal pH is 6.5–7.5 [38]. Fisogatinib is, therefore, fully protonated and ionized in the human stomach, while only partially in the mouse stomach. The combination of partial ionization and low solubility probably led to incomplete dissolution of fisogatinib in the mouse stomach. In addition, subsequent precipitation in the intestines is likely, leading to a presumably incomplete absorption in mice. Unfortunately, no intravenous data for fisogatinib in mice were available to determine the absolute F. Nevertheless, the effect of pK_a_ on the ionized fraction in the stomach was incorporated in the model of fisogatinib by decreasing the oral bioavailability in case of less-ionized fisogatinib. First, the fraction of ionized fisogatinib in the stomach for both humans and mice was calculated using Equations (3) and (4):

(3) $$fBH^{+}=\frac{1}{1+10^{pH-pKa}}$$

where *fBH^+^* is the fraction of ionized fisogatinib in the stomach for human or mouse. The average *pH* of the stomach in humans (1.75) and mice (3.5) was used as well as the *pK_a_* of fisogatinib (3.79). Secondly, the relative oral bioavailability in humans was calculated:

(4) $$F_{human}=F_{mouse}\;\cdot \;\frac{fBH_{\;human}^{+}}{fBH_{\;mouse}^{+}}$$

where *F_human_* and *F_mouse_* represent the relative oral bioavailability in humans and mice, respectively.

**Table 3:** Drug properties of lorlatinib, brigatinib, ribociclib and fisogatinib.

	Lorlatinib [39,40]	Brigatinib [40,41]	Fisogatinib [26,40]	Ribociclib [40,42]
**Primary enzymes**	CYP3A4, UGT1A4	CYP3A4, CYP2C8	CYP3A4	CYP3A4, several phase-2 enzymes
**Elimination**	With feces ~41% (~9% unchanged), with urine ~48% (mostly as metabolite)	With feces, ~65% (~41% unchanged), with urine ~25% (~86% unchanged)	NA	With feces ~69% (~17% unchanged), with urine ~23% (~12% unchanged)
**Protein binding**	66%	91%	NA	70%
**Volume of distribution (L)**	390	307	NA	1090
**pKa**	5.71 (basic)	8.54 (basic)	3.79 (basic)	8.87 (basic)
**Water solubility (mg/mL)**	0.108	0.022	0.004	0.231
**LogP**	1.63	5.17	3.86	2.38
**Molecular mass (g/mol)**	406.4	584.1	503.4	434.5

### 4.4. Simulations

In total, 500 simulations with 50 individuals were performed for the human population PK models [20,21,23]. For extrapolated human population PK models, the typical curve for 50 individuals was simulated. The human population of 50 individuals was randomly generated with an average weight of 70 kg, standard deviation of 17 kg and no weights under 50 kg based on the populations reported in the articles on human population PK models. Standard human doses of 100, 180, 600 and 600 mg were used for lorlatinib, brigatinib, ribociclib and fisogatinib, respectively. We chose to use simulated human data rather than observations from clinical studies so that the same populations could be compared for human and extrapolated models, making the models more comparable. In the simulations of the human models from literature, parameter precision was not included, but we retained the within- and between-subject variability parameters to observe whether the predictions of the extrapolated mouse models described at least part of the human population if not the median. All the variation in the simulation of the extrapolated mouse models was due to covariates within the randomly generated human population. Covariates in the human models from literature that were not available in the extrapolated mice models were set to average population values reported in the relevant article. In the case of extrapolated mouse models where model properties were omitted, both the extrapolations of the final mouse model and the optimized mouse models for mouse-to-human translation were simulated to evaluate how these properties influenced the extrapolation.

### 4.5. Comparison of Model-Derived AUC_inf_, C_max_ and PK profiles

The area under the plasma concentration–time curve from 0 to infinite time (AUC_inf_) was calculated by integration of the individually predicted concentration over time. Median AUC_inf_ and maximum concentration (C_max_) were calculated from the simulation output. Ratios compared to the clinical data were then determined for each extrapolated model. An acceptance criterion of a 0.5–2-fold difference in terms of exposure compared to literature models was used. Of the prediction interval curves, 80% were plotted for all simulations to visually compare PK profiles between extrapolated and human population PK models.

### 4.6. Software

Nonlinear, mixed-effects modeling was performed using NONMEM^®^ (version 7.4, ICON Development Solutions, Ellicott City, MD, USA) and Perl-speaks-NONMEM (PsN, version 5.2.6). Pirana (version 2.9.9) was used as the graphical user interface for NONMEM, and R (version 4.1.2) was used for processing the data and graphical and statistical diagnostics [43,44].

## 5. Conclusions

The human PK of lorlatinib and brigatinib were more closely approximated with the Cyp3aXAV compared to the WT mouse model using population PK modeling. We presented a method to extrapolate small-molecule anticancer and CYP3A4-metabolized compounds using PK data from the Cyp3aXAV mouse model and a population PK modeling approach which provided a within-0.5–2-fold prediction of the human exposure. Although CYP3A4 metabolism was extrapolated well, other contradicting PK properties between humans and mice that influence the PK of drugs such as fisogatinib and ribociclib resulted in predictions of human secondary PK parameters that only just missed the preset target of a 0.5–2-fold deviation. Optimizing the predictive capacity of models to predict the exposure of CYP3A4-metabolized compounds during preclinical research contributes to the reduction of the gap of uncertainty in interspecies extrapolation between preclinical and clinical research. The resulting, more accurate predictions of human PK can ultimately result in more safety and efficacy in FIH doses and higher success rates in drug development.

## Figures and Tables

**Table 1:** The median AUC_inf_ of lorlatinib, brigatinib, ribociclib and fisogatinib in the model from literature and the extrapolated models for wild-type and Cyp3aXAV mice for both the extrapolations of the final mouse models and the optimized mouse models (if applicable). Fold changes in AUC_inf_ of extrapolated models compared to literature model are presented. Abbreviations: AUC_inf_, area under the curve to infinity.

	Lorlatinib		Brigatinib		Fisogatinib		Ribociclib	
	Median AUC_inf_ (µg/mL h)	Fold Change	Median AUC_inf_ (µg/mL h)	Fold Change	Median AUC_inf_ (µg/mL h)	Fold Change	Median AUC_inf_ (µg/mL h)	Fold Change
**Literature model**	8.2 ± 2.4	-	13.2 ± 7.4	-	-	-	20.0 ± 11.2	-
	**Extrapolation of final mouse model**							
**Wild-type**	17.4 ± 2.4	2.1	29.8 ± 4.4	2.3	28.9 ± 4.0	-	21.3 ± 2.9	1.1
**Cyp3aXAV**	9.4 ± 1.3	1.1	19.2 ± 2.7	1.5	25.4 ± 3.7	-	6.4 ±0.9	0.3
	**Extrapolation of optimized mouse model (if applicable)**							
**Wild-type**	-	-	25.5 ± 3.9	1.9	28.9 ± 4.0	-	20.4 ± 2.8	1.0
**Cyp3aXAV**	-	-	13.7 ± 1.9	1.0	24.4 ± 3.7	-	6.1 ± 0.8	0.3

**Table 2:** The median C_max_ of lorlatinib, brigatinib, ribociclib and fisogatinib of the model from literature and the extrapolated models for wild-type and Cyp3aXAV mice for both the extrapolations of the final mouse models and the optimized mouse models (if applicable). Fold changes in C_max_ of extrapolated models compared to literature model are presented. Abbreviations: C_max_, maximum concentration.

	Lorlatinib		Brigatinib		Fisogatinib		Ribociclib	
	Median C_max_ (ng/mL)	Fold Change	Median C_max_ (ng/mL)	Fold Change	Median C_max_ (ng/mL)	Fold Change	Median C_max_ (ng/mL)	Fold Change
**Literature model**	310 ± 195	-	615 ± 422	-	6404 ± 3299	-	1176 ± 696	-
	**Extrapolation of final mouse model**							
**Wild-type**	632 ± 179	2.0	687 ± 124	1.1	4887 ± 829	0.8	2586 ± 390	2.2
**Cyp3aXAV**	431 ± 135	1.4	665 ± 116	1.1	2925 ± 501	0.5	1229 ± 177	1.0
	**Extrapolation of optimized mouse model (if applicable)**							
**Wild-type**	-	-	499 ± 91	0.8	6693 ± 513	1.0	2189 ± 323	1.9
**Cyp3aXAV**	-	-	470 ± 85	0.8	4021 ± 278	0.6	655 ± 97	0.6

## Data Availability

Not applicable.

## Supplementary Materials

The following supporting information can be downloaded at: https://www.mdpi.com/article/10.3390/ph15070860/s1, Table S1: Mouse characteristics; Table S2: Parameter estimates; Figure S1: Goodness of fit (GOF) plots; Figure S2: Visual predictive check (VPC) plots.

## Abbreviations

CYP	cytochromes P450
WT	wild-type
Cyp3aXAV	human CYP3A4 transgenic
PK	pharmacokinetic
ADME	absorption, distribution, metabolism and excretion
FIH	first-in-human
CL,	clearance
F	bioavailability
dOFVs	difference in objective function values
AUC_inf_	area under the plasma concentration–time curve from 0 to infinite time
C_max_	maximum concentration
EHC	enterohepatic circulation
RSE	relative standard error
