# Supplementary material for: Predictiveness of the Human-CYP3A4-Transgenic Mouse Model (Cyp3aXAV) for Human Drug Exposure of CYP3A4-Metabolized Drugs
Source: Pharmaceuticals (Basel). 2022 Jul 13;15(7):860. doi: 10.3390/ph15070860 (PMC9322370; doi:10.3390/ph15070860)
Supplement: Supplementary file 1 [file pharmaceuticals-15-00860-s001.zip › pharmaceuticals-1727797-supplementary.pdf]

Supplementary material

1. Methods

Data

All plasma concentrations generated in previous studies in mice receiving oral lorlatinib [1, 2], brigatinib [3], ribociclib [4] and fisogatinib [5] with no addition of boosting agents such as elacridar or ritonavir were included in this study, and characteristics are shown in table 1. Experiments were performed on mice of a >99% FVB genetic background with the strains wild type, Abcb1a/1b$^{-/-}$, Abcg2$^{-/-}$, Abcb1a/1b;Abcg2$^{-/-}$, Cyp3a$^{-/-}$ and Cyp3aXAV. Animals treated with lorlatinib, except for 11 males, received 10 mg/kg lorlatinib dissolved in DMSO, polysorbate80, ethanol and glucose in the dosing solution of 2%, 1.5%, 1.5% and 4.75% (v/v/v/w), respectively. The 11 males of which 3 wild type, 4 Cyp3a$^{-/-}$ and 4 Cyp3aXAV received accidentally a slightly lower lorlatinib dose of 8.3 mg/kg. Mice that received intravenous lorlatinib administration received 5 mg/kg. Animals treated with brigatinib received either 10 or 25 mg/kg brigatinib dissolved in 25mM sodium citrate buffer (pH 4.5). Animals treated with ribociclib received 20 mg/kg ribociclib dissolved in DMSO:Tween 80:10 mM HCl in water (5:5:90, v/v). Animals that received fisogatinib received 10 mg/kg fisogatinib dissolved in DMSO, polysorbate 80, ethanol, and 10 mM hydrochloric acid in the dosing solution were 2%, 1.5%, 1.5%, and 95% (v/v/v/v), respectively. Doses were administered by gavage into the stomach with a blunt needle or intravenously into the tail vein. Blood samples were taken from the tail vein at 0.125, 0.25, 0.5, 1, 2, 4, 8, 24 hours, varying per experiment. At the last sampling time mice were sacrificed, blood was taken by cardiac puncture and organs were harvested. After sample preparation concentrations in plasma were measured using a validated LC-MS/MS assay.

Structural and covariate model

To characterize the plasma PK of the different compounds in mice, data of all strains per compound were pooled and one- and two-compartment models were evaluated. To describe the absorption a first order, a mixed zero and first order, a dual first order absorption, a transit compartment and an enterohepatic circulation (EHC) were explored depending on the compound. For brigatinib also an exponential effect of dose on relative bioavailability was explored. Allometric scaling was used to include the effect of bodyweight on clearances (*Eq. 1*) and volumes of distribution (*Eq. 2*). Normalization to the median bodyweight was applied for all clearances and volumes of distribution using the following equations [6]:

$$CL = \theta_{pop\ CL} \cdot \left(\frac{WT}{median\ bodyweight}\right)^{0.75} \qquad \text{Eq. 1}$$

$$V = \theta_{pop\ V} \cdot \left(\frac{WT}{median\ bodyweight}\right)^{1} \qquad \text{Eq. 2}$$

Where *CL* and *V* are for bodyweight corrected estimates for clearance and distribution volume, respectively. θ_{pop} represents the population estimate for the concerning parameter and *WT* the individual bodyweight (gram) of the mouse. The *median bodyweight* represents the median bodyweight of the mouse population in gram.

Categorical covariates for strains were explored when considered physiologically plausible. Gender was tested for structural parameters for which a trend in between-subject variability (BSV) distribution versus covariate was apparent. Knocked-out transporters strains were tested as covariate on volumes of distribution, absorption rate, bioavailability and clearance. Knocked-out or human expressing metabolic enzyme strains were tested as covariate on bioavailability and clearance. Covariates were modeled using a proportional effect (*Eq. 3)*:

$$\theta_{pop.cov} = \theta_{pop} \cdot \theta_{cov}{}^{\mathrm{COV}} \qquad \text{Eq. 3}$$

Where $\theta_{pop.cov}$ represents the parameter for the population with this covariate, θ_{pop} the typical value for the population parameter, θ_{cov} the estimate for the covariate effect and COV is a binary covariate.

Stochastic model

BSV was estimated using an exponential error model (*Eq. 4*):

$$\theta_i = \theta_{pop} \cdot \exp(\eta_i) \qquad \text{Eq. 4}$$

Where θ_i represents the parameter estimate for individual *i*, θ_{pop} the typical population parameter estimate and η_i the BSV effect for individual *i* with a distribution following $N(0, \omega^2)$.

Residual unexplained variability (RUV) was described using a proportional error model (*Eq. 5*):

$$C_{\mathrm{obs},ij} = C_{\mathrm{pred},ij} \cdot \left(1 + \varepsilon_{\mathrm{prop},ij}\right) \qquad \text{Eq. 5}$$

Where $C_{\mathrm{obs,ij}}$ represents the observation for individual *i* and measurement *j*, $C_{\mathrm{pred,ij}}$ represents the prediction and $\varepsilon_{\mathrm{prop,ij}}$ the proportional error distributed following $N(0, \sigma^2)$. A separate proportional and additive error were estimated for the brain tissue measurements.

Model selection and evaluation

Models were evaluated by assessing goodness-of-fit (GOF) plots, visual predictive check (VPC) plots, change in objective function value (dOFV), ETA distribution and successful minimizations. The VPCs 10th, 50th and 90th percentile of the observations and simulations (n=500) were visually compared. dOFVs, following a chi-squared distribution, were considered significant for hierarchical models when <-6.64 (p<0.01, 1 degree of freedom) or <-9.21 (p<0.01, 2 degrees of freedom). ETA distributions were checked for a normal distribution and potential covariate effects. Parameter estimate precisions were assessed using sampling importance resampling (SIR) [7].

Model-derived AUC, C_{max} and T_{max}

The AUC_{inf} was calculated using a dummy compartment to integrate the individually predicted concentration over time. C_{max} and T_{max} were estimated by using an integration function.

Software

Nonlinear mixed-effects modeling was performed using NONMEM® (version 7.4, ICON Development Solutions, Ellicott City, MD, USA) and Perl-speaks-NONMEM (PsN, version 5.2.6). Pirana (version 2.9.9) was used as the graphical user interface for NONMEM and R (version 4.1.2) was used for processing the data and graphical and statistical diagnostics [8, 9].

2. Results

A total of 658 (of which 112 intravenous), 366, 414 and 270 plasma concentrations from 94, 61, 71 and 49 mice were modelled using a compartmental population PK modeling approach for lorlatinib, brigatinib, ribociclib and fisogatinib, respectively (table 1). There were no concentrations below the limit of quantification. This mouse population consisted of the strains wild type, Abcb1a/1b$^{-/-}$, Abcg2$^{-/-}$, Abcb1a/1b;Abcg2$^{-/-}$, Cyp3a$^{-/-}$ and Cyp3aXAV mice.

Compartmental population PK models

The compartmental models that best fitted the data are described below under the final mouse models. Although some model features led to significant model improvements for ribociclib and brigatinib in mice, these turned out to be redundant model properties for the extrapolation to human and were omitted for human extrapolation and models were re-estimated. Omitted model properties were properties that could not be scaled using allometric scaling and made little to no sense when unscaled. Resulting in the optimized mouse models for mouse-to-human translation.

Lorlatinib *[10]*

Final mouse model: A two-compartment model with a first order elimination best described the observed plasma concentration-time profiles of the pooled mice population receiving lorlatinib. Allometric scaling was used to describe the total body size effect on all clearance (*Eq. 1*) and distribution (*Eq. 2*) parameters. Visual inspection of the concentration-time curves suggested a biphasic absorption. A dual first order absorption resulted in the best fit with a dOFV of -93.6 (2 degrees of freedom, p<0.005), where 29% (RSE, 8%) of the dose was absorbed with a fast absorption rate (k_{a1} = 5.13 h^{-1} [RSE, 15%]) and 71% (RSE, 8%) of the dose was absorbed with a slow absorption rate (k_{a2} = 0.47 h^{-1} [RSE, 8%]). The final parameter estimates are summarized in table 2, lorlatinib.

Optimized mouse model: Not applicable because of no redundant model properties.

Brigatinib

Final mouse model: A two-compartment model with a first order elimination best described the observed plasma concentration-time profiles of the pooled mice population receiving brigatinib. Allometric scaling was used to describe the total body size effect on all clearance (*Eq. 1*) and distribution (*Eq. 2*) parameters. Nonlinear dose effects were observed in the concentration-time curves of brigatinib 10 and 25 mg/kg doses. An exponential effect of dose (*Eq. 6*) on bioavailability of 0.297 (RSE, 15%) was estimated resulting in a dOFV of -23.6 (1 degrees of freedom, p<0.005):

$$TVBB = COVBB \cdot (0 + \left(\frac{Dose}{1000}\right)^{0.297}) \qquad \text{Eq. 6}$$

Where *TVBB* represents the typical value of bioavailability and *COVBB* the strain covariate dependent bioavailability. The final parameter estimates are summarized in table 2, brigatinib (final mouse model).

Optimized mouse model: A two-compartment model with a first order elimination. Allometric scaling was used to describe the total body size effect on all clearance (*Eq. 1*) and distribution (*Eq. 2*) parameters. The final parameter estimates are summarized in table 2, brigatinib (optimized mouse model).

Fisogatinib

Final mouse model: A one-compartment model with a first order elimination best described the observed plasma concentration-time profiles of the pooled mice population receiving fisogatinib. The absorption was best described with transit compartments resulting in a dOFV of -215.6 (2 degrees of freedom, p<0.005) [11]. Allometric scaling was used to describe the total body size effect on all clearance (*Eq. 1*) and distribution (*Eq. 2*) parameters. The final parameter estimates are summarized in table 2, fisogatinib (final mouse model).

Optimized mouse model: Not applicable because of no redundant model properties.

Ribociclib

Final mouse model: A two-compartment model with a first order elimination best described the observed plasma concentration-time profiles of the pooled mice population receiving ribociclib. Allometric scaling was used to describe the total body size effect on all clearance (*Eq. 1*) and distribution (*Eq. 2*) parameters. An enterohepatic circulation (*Eq. 7*) significantly improved the model resulting in a dOFV of -45 (3 degrees of freedom, p<0.005) with estimates of 51.6% (RSE, 10%) for the fraction of the elimination constant to bile (FG), 2.8 hours (RSE, 10%) for the time until bile release (MTIME) and 3.6 hours (RSE, 11%) for the duration of bile release (TAU). The enterohepatic circulation was described in the model as follows (*Eq. 7*):

$$FLAG = MPAST(1) - MPAST(2) \qquad \text{Eq. 7}$$

$$\frac{dA_{depot}}{dt} = -k_a \cdot A_{depot} + \left(\frac{A_{bile}}{TAU}\right) \cdot FLAG$$

$$\frac{dA_{central}}{dt} = k_a \cdot A_{depot} + k_{42} \cdot A_{peripheral} - \Big(k_{42} + \big((1 - FG) \cdot k_e\big) + (FG \cdot k_e)\Big) \cdot A_{central}$$

$$\frac{dA_{bile}}{dt} = (FG \cdot k_e) \cdot A_{central} - \left(\frac{A_{bile}}{TAU}\right) \cdot FLAG$$

$$\frac{dA_{peripheral}}{dt} = k_{24} \cdot A_{central} - k_{42} \cdot A_{peripheral}$$

Where *A* represents the amount in either the depot, central, bile or peripheral compartment. k_a, k_e, k_{24} and k_{42} represent the absorption constant, elimination constant and the intercompartmental distribution

constants, respectively. The change-point method was used to model the bile release where *FLAG* is zero until and one during bile release and *MPAST(1)* and *MPAST(2)* are the model event times (MTIME). The final parameter estimates are summarized in table 2, ribociclib (final mouse model).

Optimized mouse model: A two-compartment model with a first order elimination. Allometric scaling was used to describe the total body size effect on all clearance (*Eq. 1*) and distribution (*Eq. 2*) parameters. The final parameter estimates are summarized in table 2, ribociclib (final mouse model).

3. Figures and tables

A. Table S1: characteristics
B. Table S2: parameter estimates
C. Figure S1: Goodness of fit (GOF) plots
D. Figure S2: Visual predictive check (VPC) plots

A. Table S1: Characteristics of the mouse population per compound used for the models

Strains	Wild type	Abcb1a/1b$^{-/-}$	Abcg2$^{-/-}$	Abcb1a/1b; Abcg2$^{-/-}$	Cyp3a$^{-/-}$	Cyp3aXAV	All mice
**Lorlatinib**							
No. of mice (of which IV)	36 (6)	6	6	18	22 (5)	22 (5)	110 (16)
No. of PK plasma samples (of which IV)	223 (42)	36	36	102	143 (35)	145 (35)	685 (112)
No. of PK plasma samples/mice (mean ± SD)	6.19 ± 0.71	6 ± 0	6 ± 0	5.67 ± 0.49	6.5 ± 0.51	6.59 ± 0.5	6.23 ± 0.63
Weight, g (mean ± SD)	29.24 ± 4.52	26.53 ± 1.57	24.03 ± 2.04	32.01 ± 3.57	31.41 ± 7.1	35.67 ± 3.62	30.98 ± 5.52
Age, weeks (mean ± SD)	10.64 ± 2.37	10.5 ± 0.55	11 ± 0	11.61 ± 1.14	8.55 ± 2.28	10.82 ± 2.44	10.43 ± 2.28
Sex (n, (%))							
Male	16 (53.3%)	0 (0%)	0 (0%)	6 (33.3%)	10 (58.8%)	12 (70.6%)	60 (54.5%)
Female	14 (46.7%)	6 (100%)	6 (100%)	12 (66.7%)	7 (41.2%)	5 (29.4%)	50 (45.5%)
**Brigatinib**							
No. of mice	7	12	7	15	6	14	61
No. of PK plasma samples	42	72	42	90	36	84	366
No. of PK plasma samples/mice (mean ± SD)	6 ± 0	6 ± 0	6 ± 0	6 ± 0	6 ± 0	6 ± 0	6 ± 0
Weight, g (mean ± SD)	30.64 ± 2.83	33.67 ± 3.01	33.71 ± 2.78	35.55 ± 2.87	35.97 ± 3.09	32.21 ± 2.04	33.68 ± 3.14
Age, weeks (mean ± SD)	11 ± 1	11 ± 1.48	14.71 ± 0.49	12.73 ± 2.25	15 ± 0	12.57 ± 0.76	12.61 ± 1.93
Sex (n, (%))							
Male	7 (100%)	12 (100%)	7 (100%)	15 (100%)	6 (100%)	14 (100%)	61 (100%)
Female	0 (0%)	0 (0%)	0 (0%)	0 (0%)	0 (0%)	0 (0%)	0 (0%)
**Ribociclib**							
No. of mice	6	18	6	13	8	20	71
No. of PK plasma samples	36	102	36	78	48	114	414

**No. of PK plasma samples/mice (mean ± SD)**	6 ± 0	5.67 ± 0.49	6 ± 0	6 ± 0	6 ± 0	5.7 ± 0.47	5.83 ± 0.38
**Weight, g (mean ± SD)**	26.77 ± 4	29.5 ± 3.6	26 ± 1.42	31.03 ± 2.37	31.86 ± 2.99	25.39 ± 3.24	28.36 ± 3.93
**Age, weeks (mean ± SD)**	14.67 ± 0.52	12.56 ± 2.28	15.33 ± 0.52	13.85 ± 1.46	14 ± 1.07	12.5 ± 2.09	13.35 ± 1.98
**Sex (n, (%))**							
**Male**	0 (0%)	0 (0%)	0 (0%)	0 (0%)	0 (0%)	0 (0%)	0 (0%)
**Female**	6 (100%)	18 (100%)	6 (100%)	13 (100%)	8 (100%)	20 (100%)	71 (100%)
**Fisogatinib**							
**No. of mice**	6	12	6	6	7	12	49
**No. of PK plasma samples**	30	66	30	36	42	66	270
**No. of PK plasma samples/mice (mean ± SD)**	5 ± 0	5.5 ± 0.52	5 ± 0	6 ± 0	6 ± 0	5.5 ± 0.52	5.51 ± 0.51
**Weight, g (mean ± SD)**	35.23 ± 3.19	35.33 ± 4.44	31.93 ± 1.12	32 ± 5.19	33.89 ± 3.61	31.84 ± 4.06	33.43 ± 4.05
**Age, weeks (mean ± SD)**	13 ± 0	11.5 ± 0.67	13 ± 0	10.67 ± 1.03	12.29 ± 0.49	11.75 ± 1.36	11.94 ± 1.11
**Sex (n, (%))**							
**Male**	6 (100%)	12 (100%)	6 (100%)	6 (100%)	7 (100%)	12 (100%)	49 (100%)
**Female**	0 (0%)	0 (0%)	0 (0%)	0 (0%)	0 (0%)	0 (0%)	0 (0%)

B. Table S2: Parameter estimates of the compartmental population PK model of lorlatinib, brigatinib, ribociclib and fisogatinib in mice. RSE, relative standard error.

Lorlatinib

**Parameter**		**Estimate**	**RSE**	**Shrinkage**
Oral bioavailability	BB	0.847	4%	-
Fraction of dose (depot 1)	F1	0.289	8%	-
Fraction of dose (depot 2)	1-F1	0.711	-	-
Absorption rate constant (depot 1)	k_{a1}	5.13 h^{-1}	15%	-
Absorption rate constant (depot 2)	k_{a2}	0.473 h^{-1}	8%	-
Volume of distribution (central)	V_c	0.0227 L	11%	-
Clearance	CL	0.0141 L/h	3%	-
Volume of distribution (peripheral)	V_p	0.0328 L	6%	-
Intercompartmental clearance	Q	0.0504 L/h	7%	-
**Covariates**				
Oral bioavailability for Cyp3aXAV	BB	x0.71	7%	-
Absorption rate constant (depot 2) for Abcb1a/1b;Abcg2$^{-/-}$	ka_2	x0.64	11%	-
Clearance for Cyp3aXAV	CL	x1.34	5%	-
**Between-subject variability**				
Variation in oral bioavailability	-	10.2%	33%	37%
Variation in absorption rate constant (depot 2)	-	25.7%	31%	43%
Variation in volume of distribution (central)	-	46.5%	20%	14%
Variation in clearance	-	9.4%	24%	36%
**Residual unexplained variability**				
Proportional residual error	-	17.2%	10%	12%

Brigatinib (final mouse model)

Parameter		Estimate	RSE	Shrinkage
Absorption rate constant	k_a	0.717 h^{-1}	13%	-
Volume of distribution (central)	V_c/F	0.0244 L	40%	-
Clearance	CL/F	0.0155 L/h	7%	-
Volume of distribution (peripheral)	V_p/F	0.0648 L	15%	-
Intercompartmental clearance	Q/F	0.474 L/h	37%	-
**Covariates**				
Oral bioavailability for Abcb1a/1b; Abcg2$^{-/-}$	BB	x0.48	7%	-
Oral bioavailability for Cyp3a$^{-/-}$	BB	x1.35	7%	-
Oral bioavailability for exponential dose effect	BB	0.297	15%	-
Absorption rate constant for Abcb1a/1b; Abcg2$^{-/-}$	k_a	x4.42	27%	-
Absorption rate constant for Abcg2$^{-/-}$	k_a	x1.5	22%	-
Clearance for Abcb1a/1b; Abcg2$^{-/-}$	CL	x0.48	8%	-
Clearance for Cyp3aXAV	CL	x1.36	13%	-
**Between-subject variability**				
Variation in oral bioavailability	-	14.3%	32%	25%
Variation in absorption rate constant	-	72.9%	22%	10%
Variation in volume of distribution (central)	-	27.3%	50%	67%
Variation in clearance	-	14.3%	38%	41%
**Residual unexplained variability**				
Proportional residual error	-	17.9%	14%	16%

Brigatinib (optimized mouse model)

Parameter		Estimate	RSE	Shrinkage

Absorption rate constant	k_a	0.91 h^{-1}	12%	-
Volume of distribution (central)	V_c/F	0.0525 L	23%	-
Clearance	CL/F	0.0175 L/h	5%	-
Volume of distribution (peripheral)	V_p/F	0.0766 L	11%	-
Intercompartmental clearance	Q/F	0.777 L/h	36%	-
**Covariates**				
Oral bioavailability for Cyp3a$^{-/-}$	BB	x1.4	6%	-
Absorption rate constant for Abcb1a/1b; Abcg2$^{-/-}$	k_a	x3.65	34%	-
Volume of distribution (peripheral) for Abcb1a/1b$^{-/-}$	V_p	x1.71	10%	-
Clearance for Abcb1a/1b; Abcg2$^{-/-}$	CL	x0.585	7%	-
Clearance for Cyp3aXAV	CL	x2.06	9%	-
**Between-subject variability**				
Variation in absorption rate constant	-	77.4%	30%	15%
Variation in volume of distribution (central)	-	93.2%	45%	16%
Variation in clearance	-	11.5%	67%	43%
**Residual unexplained variability**				
Proportional residual error	-	17.6%	15%	16%

Ribociclib (final mouse model)

**Parameter**		**Estimate**	**RSE**	**Shrinkage**
Absorption rate constant	k_a	0.382 h^{-1}	6%	-
Volume of distribution (central)	V_c/F	0.0255 L	11%	-
Clearance	CL/F	0.104 L/h	6%	-
Volume of distribution (peripheral)	V_p/F	0.113 L	13%	-

Intercompartmental clearance	Q/F	0.0412 L/h	17%	-
Eliminated fraction to bile	FG	51.6%	10%	-
Time to bile release	MTIME	2.8 h	10%	-
Duration of bile release	TAU	3.6 h	11%	-
**Covariates**				
Oral bioavailability for Abcb1a/1b$^{-/-}$ and Abcb1a/1b; Abcg2$^{-/-}$	BB	x1.43	8%	-
Oral bioavailability for Cyp3a$^{-/-}$	BB	x1.21	8%	-
Absorption rate constant for Abcb1a/1b$^{-/-}$	k_a	x0.688	13%	-
Absorption rate constant for Abcb1a/1b; Abcg2$^{-/-}$	k_a	x0.539	9%	-
Volume of distribution (peripheral) for Abcg2$^{-/-}$	V_p	x0.151	22%	-
Clearance for Cyp3aXAV	CL	x3.46	8%	-
**Between-subject variability**				
Variation in oral bioavailability wild type	-	41.1%	21%	52%
Variation in oral bioavailability other strains	-	17.4%	19%	26%
Variation in absorption rate constant	-	29.1%	16%	10%
Variation in volume of distribution (central)	-	35.4%	21%	38%
Variation in time to bile release	-	49.6.2%	21%	37%
**Residual unexplained variability**				
Proportional residual error	-	13.6%	11%	52%

Ribociclib (optimized mouse model)

Parameter		Estimate	RSE	Shrinkage

Absorption rate constant	k_a	0.393 h^{-1}	10%	-
Volume of distribution (central)	V_c/F	0.0216 L	19%	-
Clearance	CL/F	0.0888 L/h	5%	-
Volume of distribution (peripheral)	V_p/F	0.19 L	10%	-
Intercompartmental clearance	Q/F	0.115 L/h	15%	-
**Covariates**				
Oral bioavailability for Abcb1a/1b; $Abcg2^{-/-}$	BB	x0.725	10%	-
Oral bioavailability for $Cyp3a^{-/-}$	BB	x1.3	8%	-
Oral bioavailability for Cyp3aXAV	BB	x0.299	8%	-
Volume of distribution (peripheral) for $Abcg2^{-/-}$	V_p	x0.57	20%	-
Clearance for $Abcb1a/1b^{-/-}$	CL	x0.654	8%	-
Clearance for Abcb1a/1b; $Abcg2^{-/-}$	CL	x0.434	10%	-
**Between-subject variability**				
Variation in oral bioavailability	-	23.6%	25%	9%
Variation in absorption rate constant	-	38.2%	29%	14%
Variation in volume of distribution (central)	-	10.3%	232%	88%
**Residual unexplained variability**				
Proportional residual error	-	20%	12%	16%

Fisogatinib (final mouse model)

**Parameter**		**Estimate**	**RSE**	**Shrinkage**
Mean transit time	MTT	0.0385 h	10%	-
Amount of transit compartments	N	1.33	45%	-
Absorption rate constant	k_a	3.24 h^{-1}	13%	-

Volume of distribution (central)	V_c/F	0.0308 L	11%	-
Clearance	CL/F	0.0618 L/h	4%	-
Volume of distribution (peripheral)	V_p/F	0.0548 L	8%	-
Intercompartmental clearance	Q/F	0.109 L/h	7%	-
**Covariates**				
Oral bioavailability for Abcb1a/1b; Abcg2$^{-/-}$	BB	x1.32	5%	-
Oral bioavailability for Cyp3aXAV	BB	x0.566	10%	-
Absorption rate constant for Abcg2$^{-/-}$	k_a	x1.8	16%	-
Volume of distribution (peripheral) for Abcb1a/1b; Abcg2$^{-/-}$	V_p	x1.42	10%	-
Clearance for Cyp3a$^{-/-}$	CL	x0.724	5%	-
Clearance for Cyp3aXAV$^{-/-}$	CL	x0.612	6%	-
**Between-subject variability**				
Variation in oral bioavailability	-	17.7%	23%	5%
Variation in mean transit time	-	36.6%	41%	23%
Variation in absorption rate constant	-	35.2%	24%	7%
Variation in volume of distribution (peripheral)	-	11.2%	114%	54%
**Residual unexplained variability**				
Proportional residual error	-	9.4%	14%	27%

C. Figure S1 GOF plots

Lorlatinib

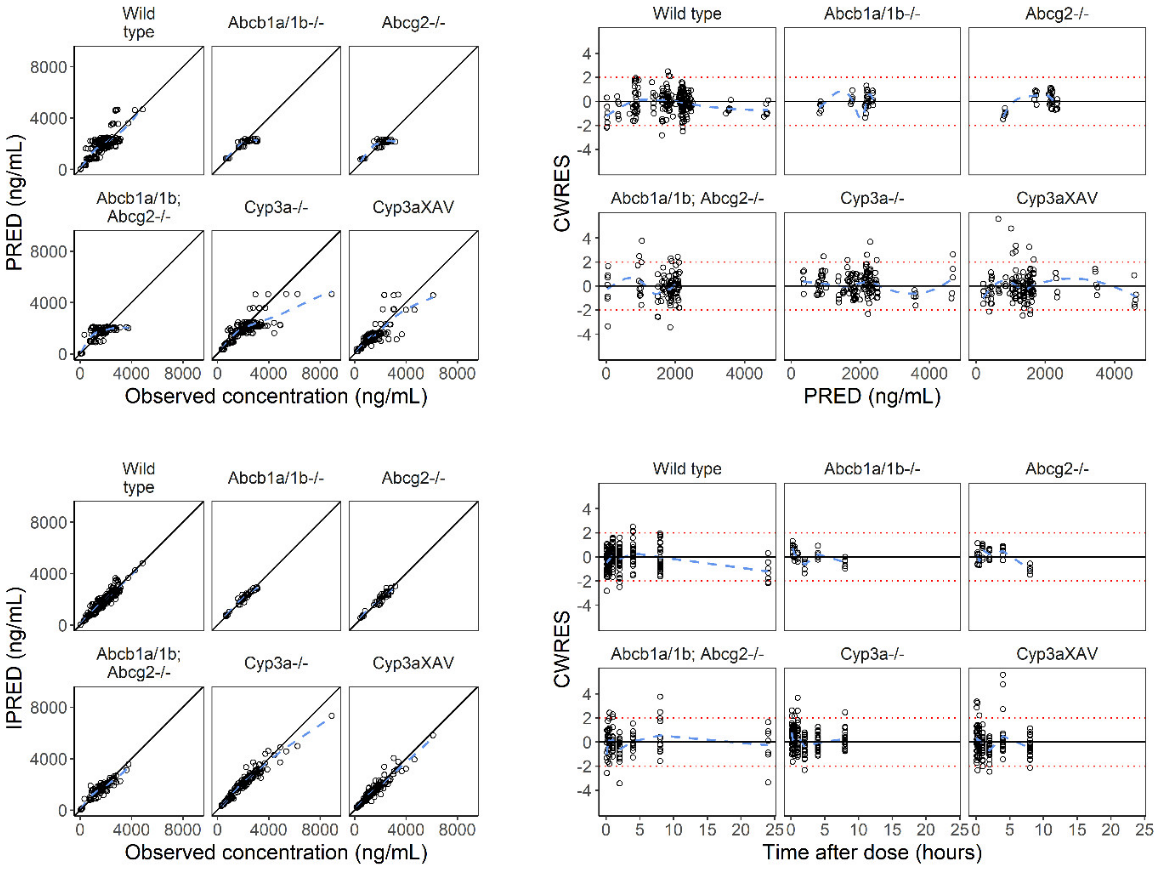

Brigatinib (final mouse model)

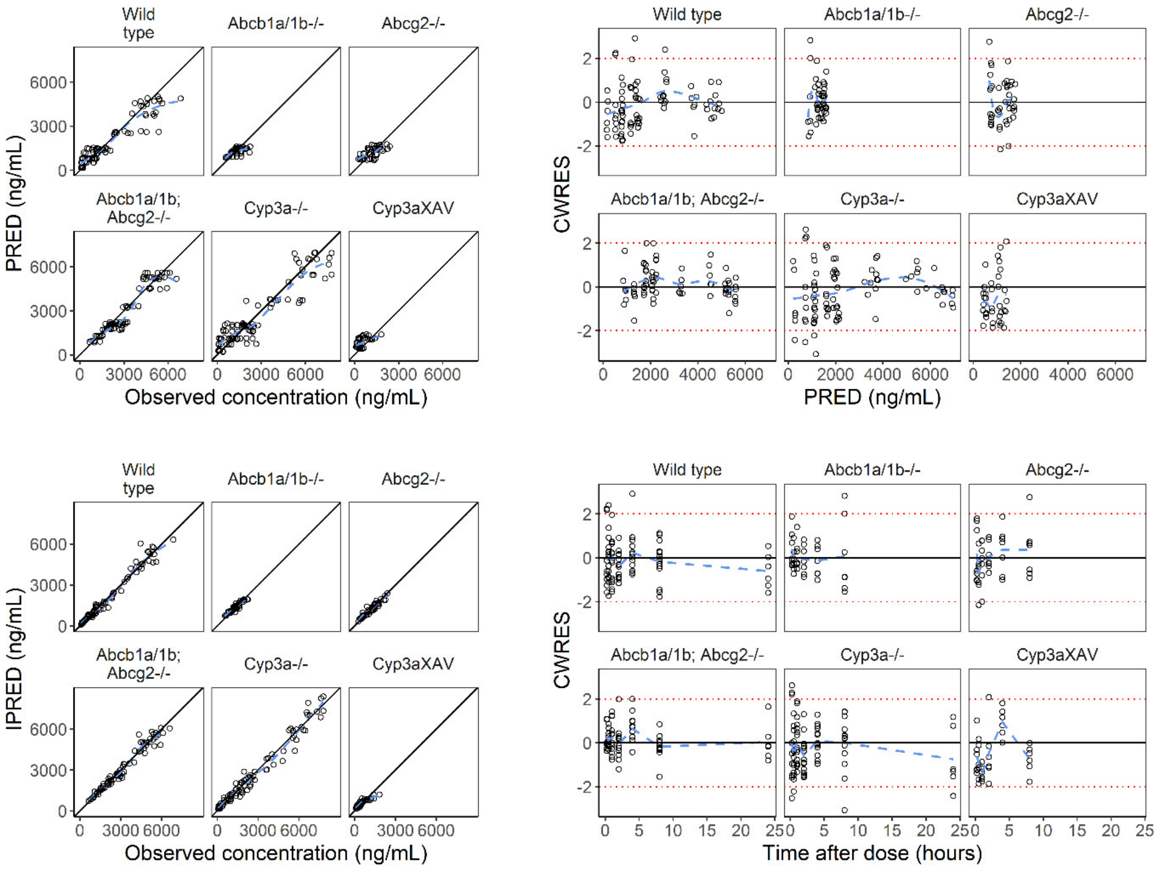

Brigatinib (optimized mouse model)

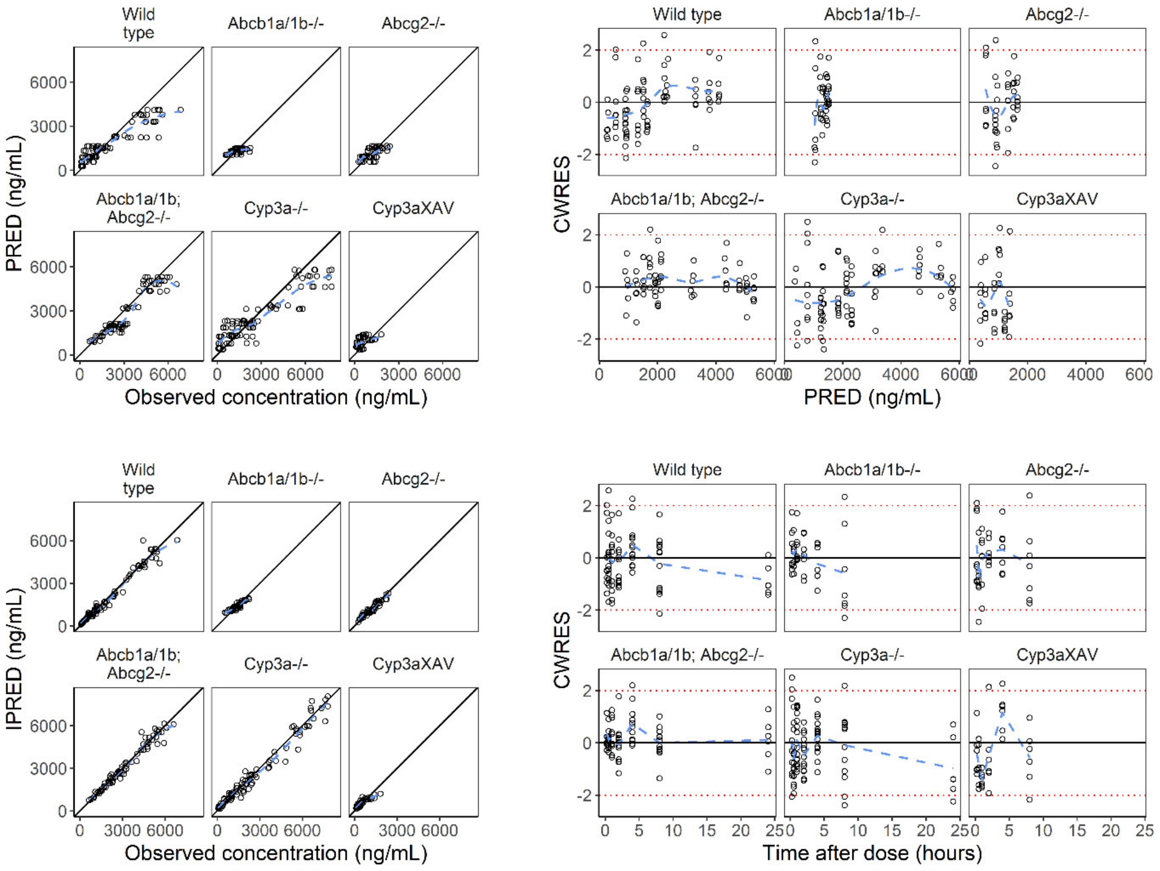

Ribociclib (final mouse model)

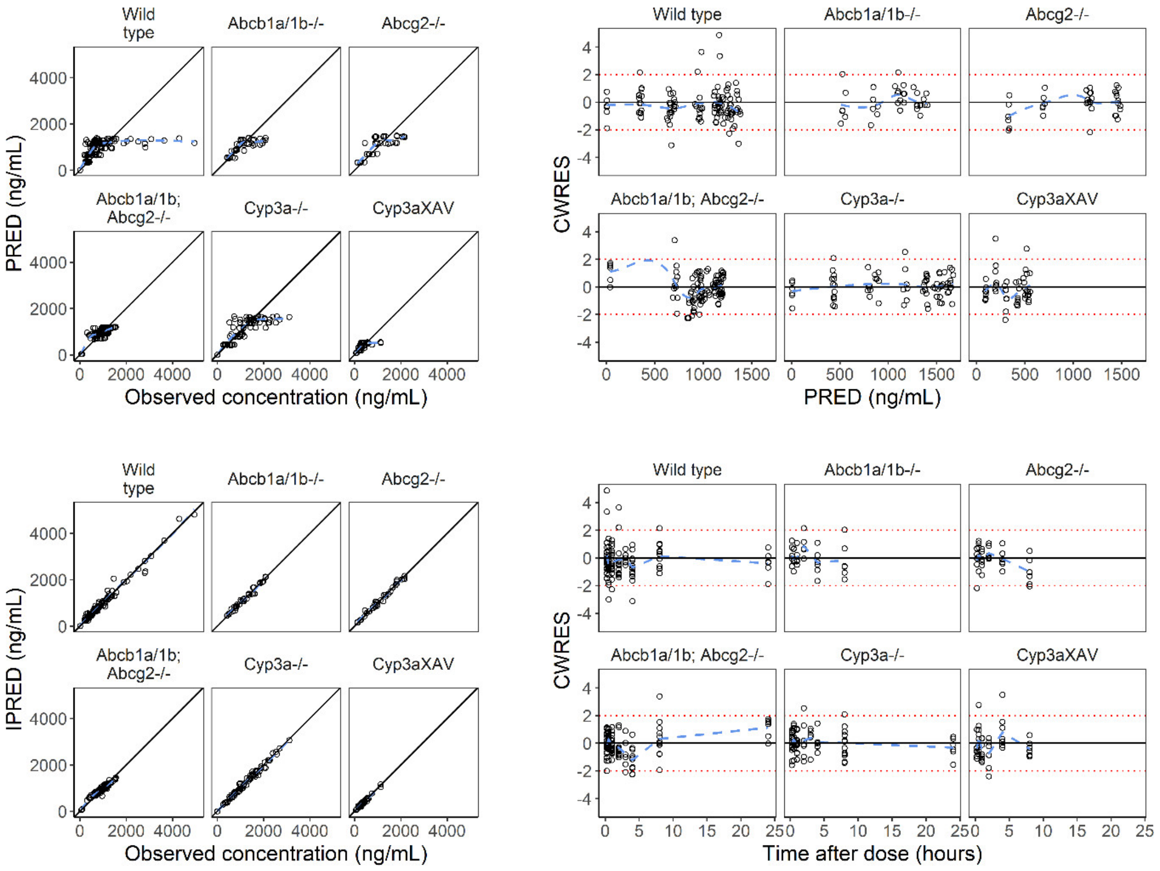

Ribociclib (optimized mouse model)

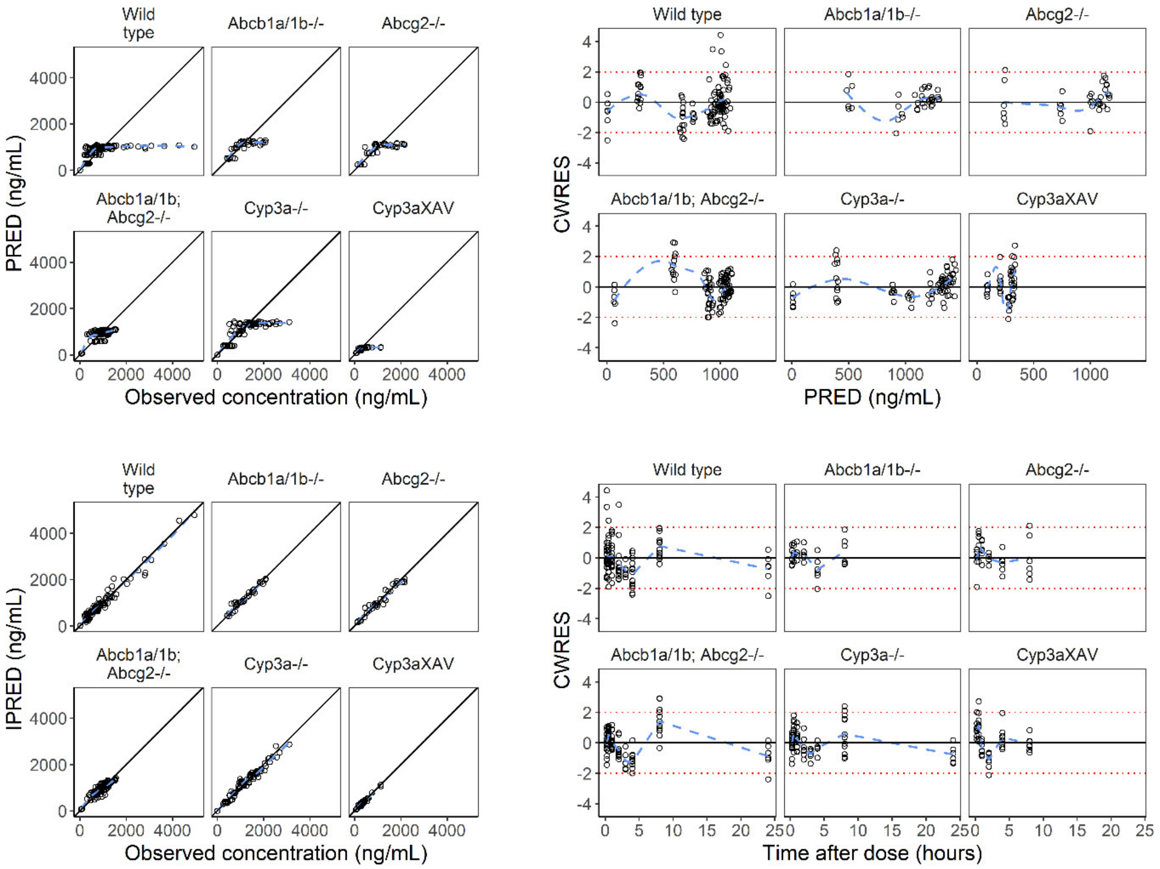

Fisogatinib (final mouse model)

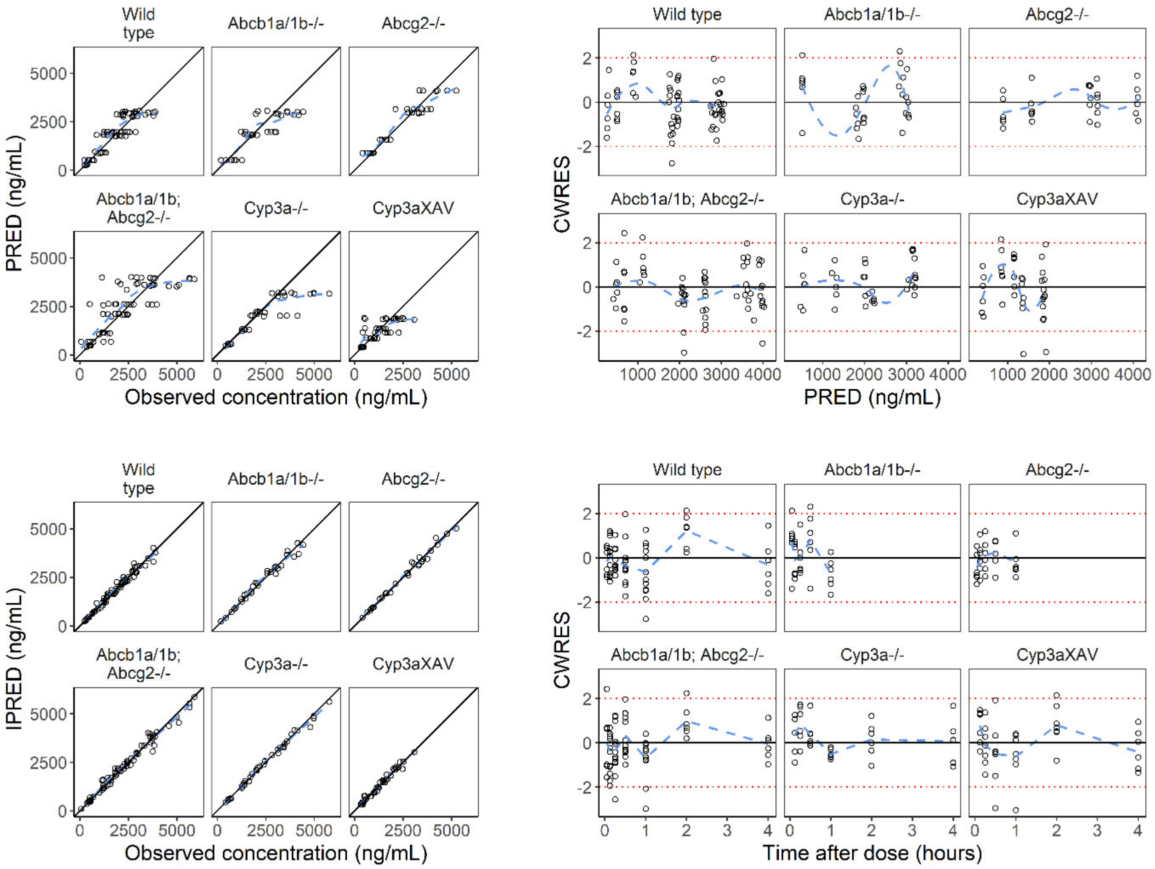

Figure S1: Goodness-of-fit plots of the pharmacokinetic models stratified per strain for plasma. Plots show individual and population predictions versus the observed concentrations and the conditional weighted residuals versus population predictions and time after administration. PRED, population predictions; IPRED, individual predictions; CWRES, conditional weighted residuals.

D. Figure S2 VPCs

Lorlatinib IV

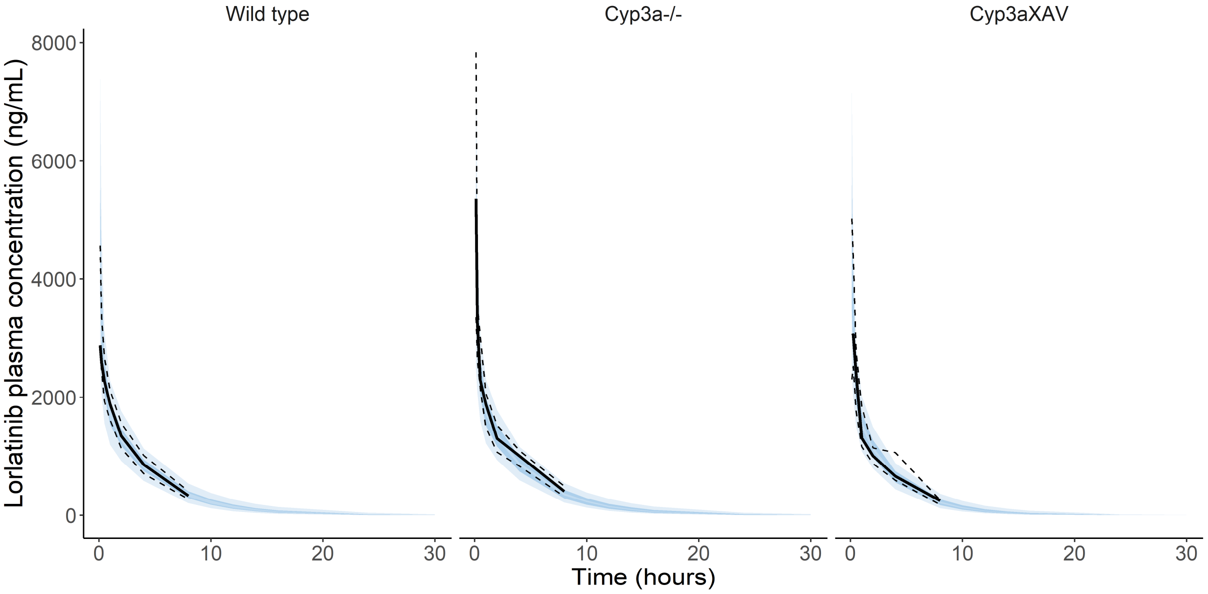

Lorlatinib oral

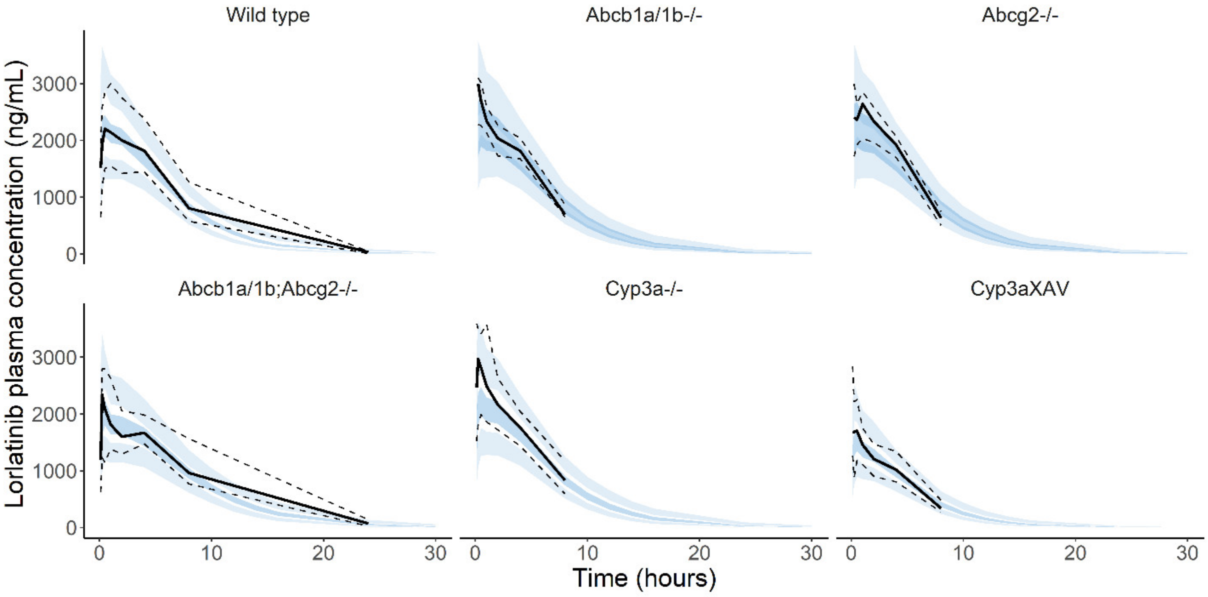

Brigatinib 10 mg (final mouse model)

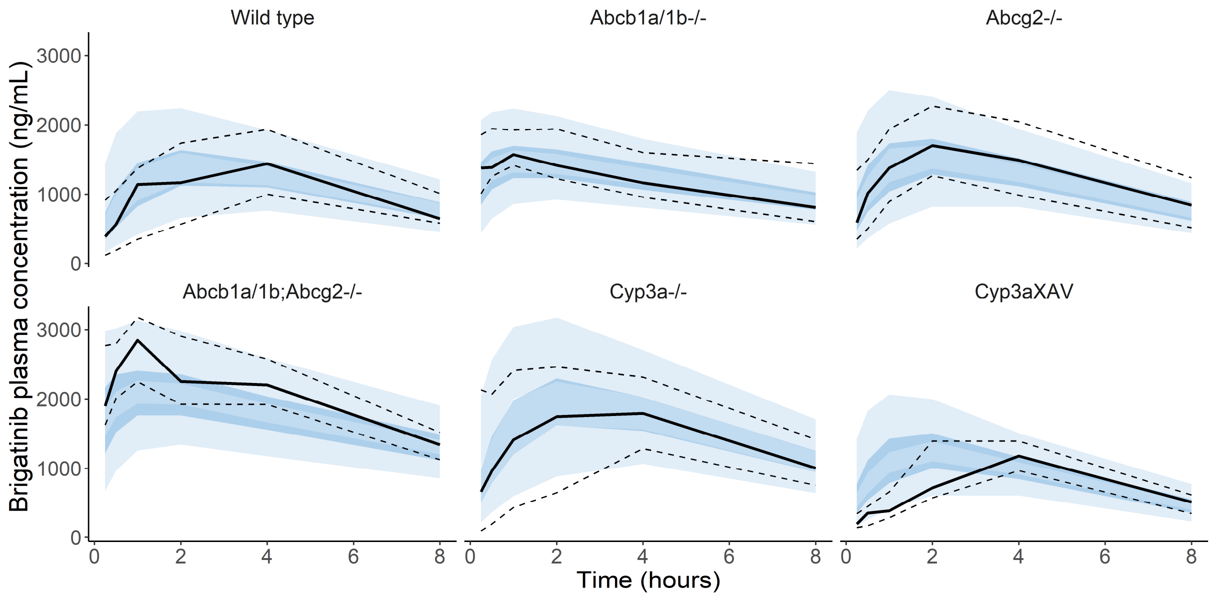

Brigatinib 25 mg (final mouse model)

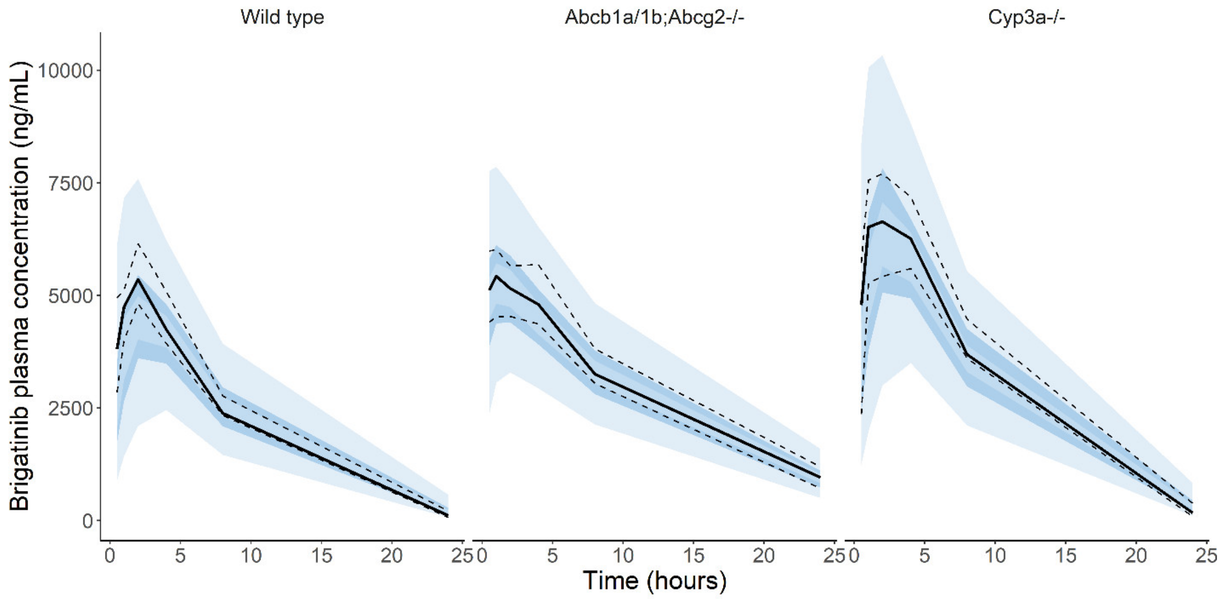

Brigatinib 10 mg (optimized mouse model)

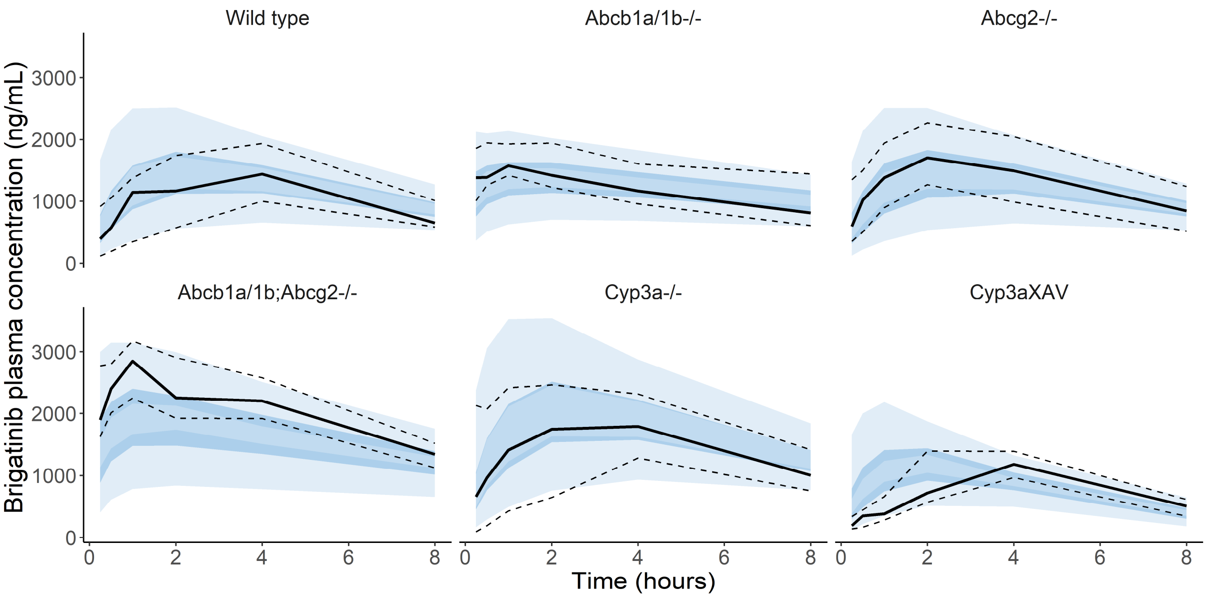

Brigatinib 25 mg/kg (optimized mouse model)

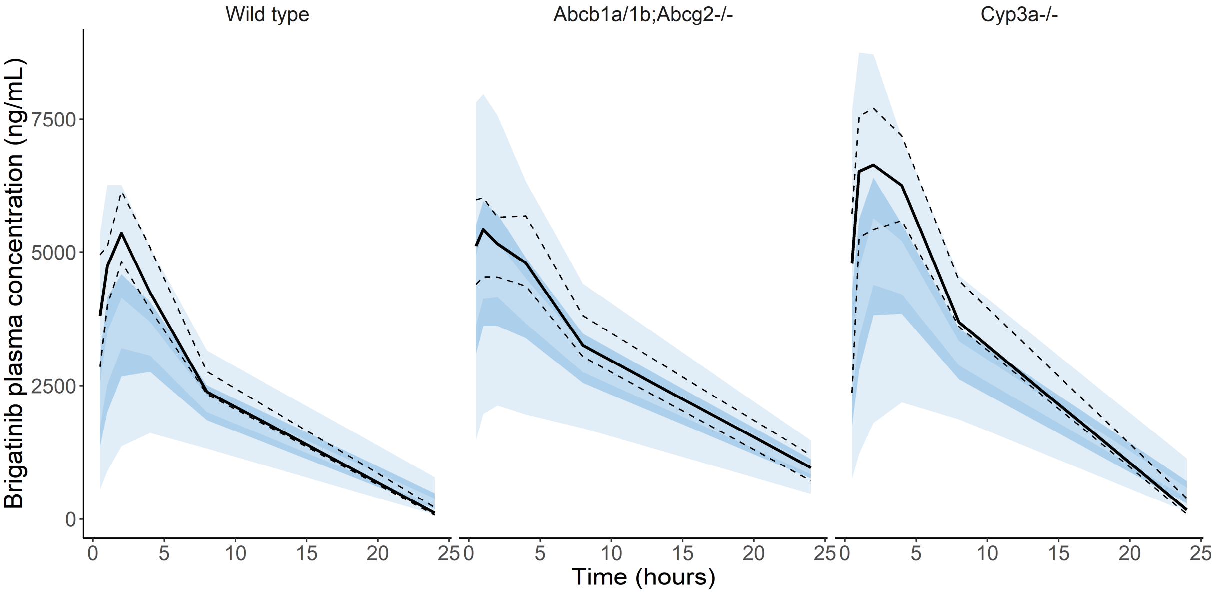

Ribociclib (final mouse model)

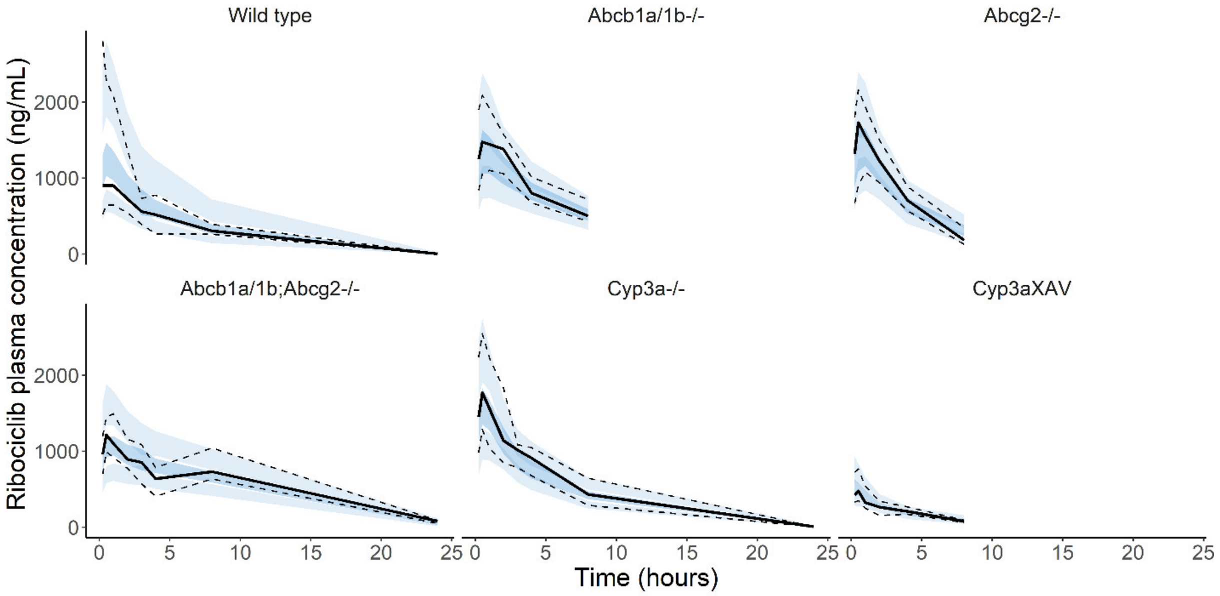

Ribociclib (optimized mouse model)

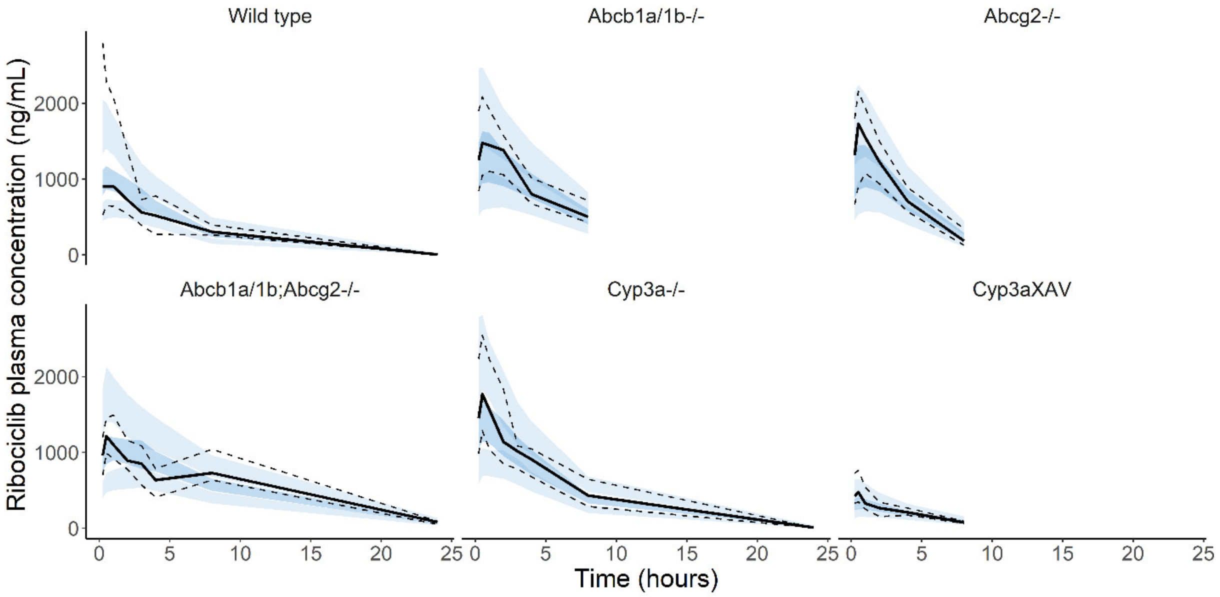

Fisogatinib (final mouse model)

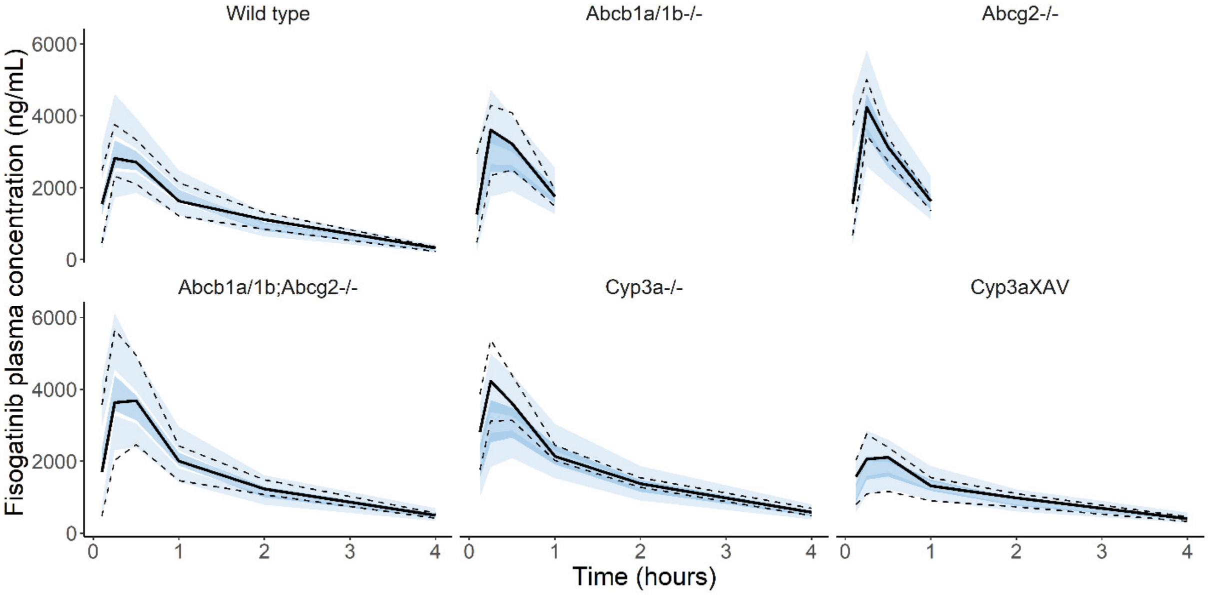

Figure S2: Visual predictive checks of the intravenous pharmacokinetic model for lorlatinib stratified per strain for plasma and the oral pharmacokinetic model for lorlatinib stratified per strain for plasma. Solid lines and dark blue areas represent the median observed values and simulated 90% CIs. Dashed lines and light blue areas represent the 10% and 90% percentiles of the observed values and 90% CIs of the simulated percentiles. Visual predictive checks consisted out of 1000 simulations each. CIs, confidence intervals.

References

1. Li, W., et al., *Oral coadministration of elacridar and ritonavir enhances brain accumulation and oral availability of the novel ALK/ROS1 inhibitor lorlatinib.* Eur J Pharm Biopharm, 2019. **136**: p. 120-130.
2. Li, W., et al., *P-glycoprotein (MDR1/ABCB1) restricts brain accumulation and cytochrome P450-3A (CYP3A) limits oral availability of the novel ALK/ROS1 inhibitor lorlatinib.* Int J Cancer, 2018. **143**(8): p. 2029-2038.
3. Li, W., et al., *P-glycoprotein and breast cancer resistance protein restrict brigatinib brain accumulation and toxicity, and, alongside CYP3A, limit its oral availability.* Pharmacol Res, 2018. **137**: p. 47-55.
4. Martínez-Chávez, A., et al., *P-glycoprotein Limits Ribociclib Brain Exposure and CYP3A4 Restricts Its Oral Bioavailability.* Mol Pharm, 2019. **16**(9): p. 3842-3852.
5. Li, W., et al., *P-glycoprotein (ABCB1/MDR1) limits brain accumulation and Cytochrome P450-3A (CYP3A) restricts oral availability of the novel FGFR4 inhibitor fisogatinib (BLU-554).* Int J Pharm, 2020. **573**: p. 118842.
6. West, G.B., J.H. Brown, and B.J. Enquist, *A general model for the origin of allometric scaling laws in biology.* Science, 1997. **276**(5309): p. 122-6.

7. Dosne, A.G., M. Bergstrand, and M.O. Karlsson, *An automated sampling importance resampling procedure for estimating parameter uncertainty.* J Pharmacokinet Pharmacodyn, 2017. **44**(6): p. 509-520.
8. Keizer, R.J., M.O. Karlsson, and A. Hooker, *Modeling and Simulation Workbench for NONMEM: Tutorial on Pirana, PsN, and Xpose.* CPT Pharmacometrics Syst Pharmacol, 2013. **2**(6): p. e50.
9. Beal, S., A. Boeckmann, and L. Sheiner, *NONMEM user guides. San Francisco: University of California, San Francisco.* 1988.
10. Damoiseaux, D., et al., *Population pharmacokinetic modelling to support the evaluation of preclinical pharmacokinetic experiments with lorlatinib.* J Pharm Sci, 2021.
11. Savic, R.M., et al., *Implementation of a transit compartment model for describing drug absorption in pharmacokinetic studies.* J Pharmacokinet Pharmacodyn, 2007. **34**(5): p. 711-26.
